# Assessing hemodynamics from the photoplethysmogram to gain insights into vascular age: a review from VascAgeNet

**DOI:** 10.1152/ajpheart.00392.2021

**Published:** 2021-12-24

**Authors:** Peter H. Charlton, Birutė Paliakaitė, Kristjan Pilt, Martin Bachler, Serena Zanelli, Dániel Kulin, John Allen, Magid Hallab, Elisabetta Bianchini, Christopher C. Mayer, Dimitrios Terentes-Printzios, Verena Dittrich, Bernhard Hametner, Dave Veerasingam, Dejan Žikić, Vaidotas Marozas

**Affiliations:** ^1^Department of Public Health and Primary Care, University of Cambridge, Cambridge, United Kingdom; ^2^Research Centre for Biomedical Engineering, University of London, London, United Kingdom; ^3^Biomedical Engineering Institute, Kaunas University of Technology, Kaunas, Lithuania; ^4^Department of Health Technologies, Tallinn University of Technology, Tallinn, Estonia; ^5^Biomedical Systems, Center for Health and Bioresources, AIT Austrian Institute of Technology, Seibersdorf, Austria; ^6^Laboratoire Analyze, Géométrie et Applications, University Sorbonne Paris Nord, Paris, France; ^7^Axelife, Redon, France; ^8^Institute of Translational Medicine, Semmelweis University, Budapest, Hungary; ^9^E-Med4All Europe, Limited, Budapest, Hungary; ^10^Research Centre for Intelligent Healthcare, Coventry University, Coventry, United Kingdom; ^11^Faculty of Medical Sciences, Newcastle University, Newcastle upon Tyne, United Kingdom; ^12^Centre de recherche et d'Innovation, Clinique Bizet, Paris, France; ^13^Institute of Clinical Physiology, CNR, Pisa, Italy; ^14^Hypertension and Cardiometabolic Unit, First Department of Cardiology, Hippokration Hospital, Medical School, National and Kapodistrian University of Athens, Athens, Greece; ^15^Redwave Medical, Gesellschaft mit beschränkter Haftung, Jena, Germany; ^16^Department of Cardiothoracic Surgery, Galway University Hospitals, Galway, Ireland; ^17^Faculty of Medicine, Institute of Biophysics, University of Belgrade, Belgrade, Serbia

**Keywords:** arterial stiffness, arteriosclerosis, atherosclerosis, blood pressure, photoplethysmography, pulse wave velocity

## Abstract

The photoplethysmogram (PPG) signal is widely measured by clinical and consumer devices, and it is emerging as a potential tool for assessing vascular age. The shape and timing of the PPG pulse wave are both influenced by normal vascular aging, changes in arterial stiffness and blood pressure, and atherosclerosis. This review summarizes research into assessing vascular age from the PPG. Three categories of approaches are described: *1*) those which use a single PPG signal (based on pulse wave analysis), *2*) those which use multiple PPG signals (such as pulse transit time measurement), and *3*) those which use PPG and other signals (such as pulse arrival time measurement). Evidence is then presented on the performance, repeatability and reproducibility, and clinical utility of PPG-derived parameters of vascular age. Finally, the review outlines key directions for future research to realize the full potential of photoplethysmography for assessing vascular age.

## INTRODUCTION

Vascular age is an emerging indicator of cardiovascular health that is indicative of cardiovascular risk, and can prompt clinical intervention (1). The function and structure of blood vessels naturally degrade with age (2). This process, known as vascular aging, includes an increase in the stiffness and diameter of the larger arteries and lengthening of the proximal aorta (3, 4). It can ultimately result in damage to the heart, kidney, and brain (1). Indicators of vascular aging have been found to be predictive of cardiovascular morbidity and all-cause mortality, such as the assessment of aortic stiffness by carotid-femoral pulse wave velocity (as assessed using applanation tonometry or vascular ultrasonography) (5). Other indicators are routinely used for diagnosis, such as the ankle-brachial index being used to diagnose peripheral arterial disease (PAD). Consequently, it is helpful to identify individuals with early vascular aging for clinical intervention (6): those whose vascular age (apparent age of the blood vessels) is greater than their chronological age (time since birth). However, many current approaches to assess vascular age are not yet widely used, in part due to the need for a trained operator and standardized measurement conditions.

**Figure 1.**
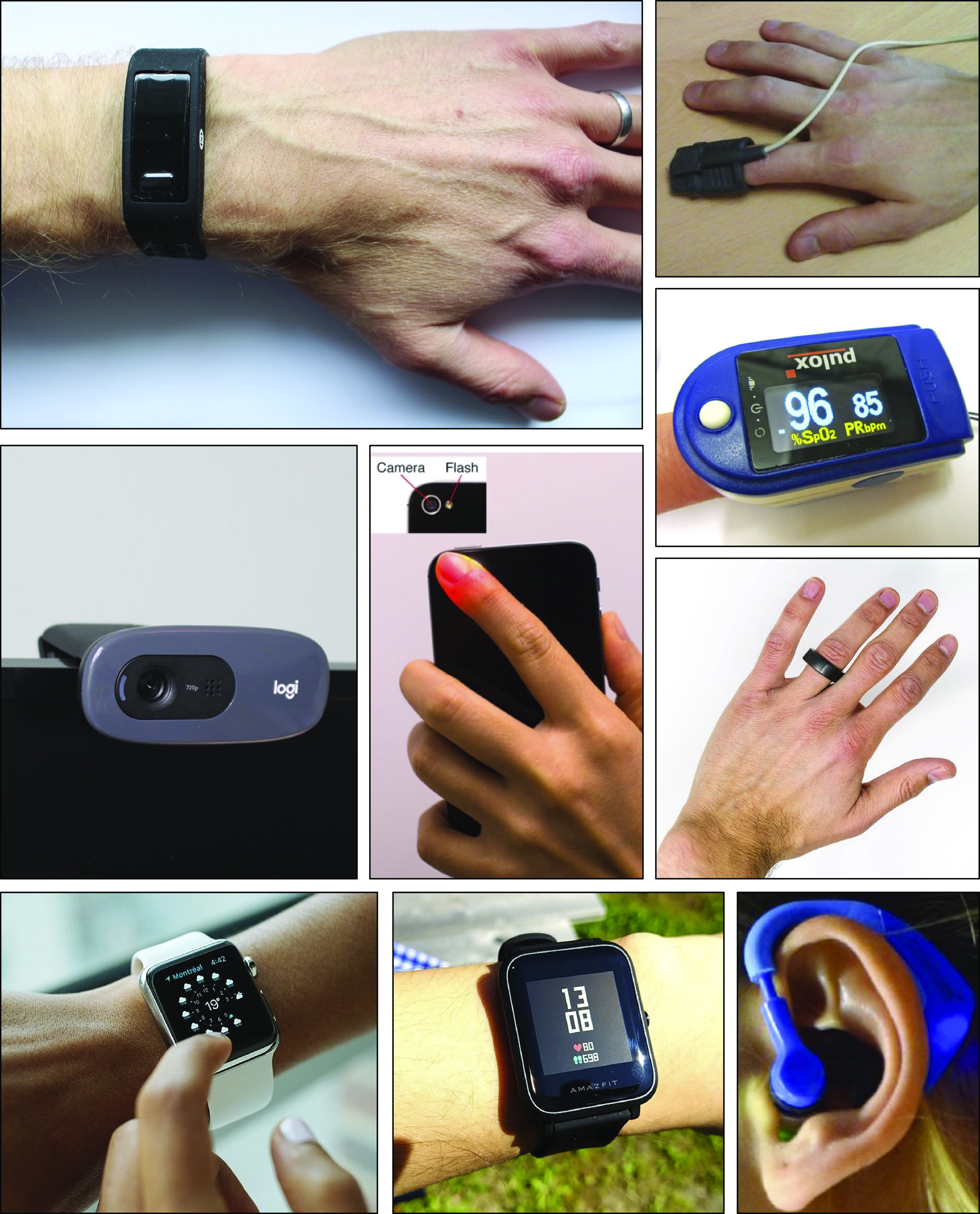
Devices for measuring the photoplethysmogram (PPG) signal. The PPG can be measured by several clinical and consumer devices, including (clockwise from *top left*): wristbands, pulse oximeters (×2), smart rings, hearables, smartwatches (×2), webcams, and smartphones. Sources (clockwise from top): P. H. Charlton, Max Health Band (“https://commons.wikimedia.org/wiki/File:Max_Health_Band.jpg”) (“https://creativecommons.org/licenses/by/4.0/” CC BY 4.0); P. H. Charlton, Wrist pulse oximeter (“https://commons.wikimedia.org/wiki/File:Wrist_pulse_oximeter.jpg”) (“https://creativecommons.org/licenses/by/4.0/” CC BY 4.0); Stefan Bellini, Pulox Pulse Oximeter (“https://commons.wikimedia.org/wiki/File:Pulox_Pulse_Oximeter.JPG”) (“https://creativecommons.org/publicdomain/zero/1.0/” CC0 1.0) M. Verch, https://flickr.com/photos/160866001@N07/32586534637/ (“https://creativecommons.org/licenses/by/2.0/” CC BY 2.0); S. Passler et al. (242) https://doi.org/10.3390/s19173641 (“https://creativecommons.org/licenses/by/4.0/” CC BY 4.0); GEEK KAZU, https://www.flickr.com/photos/152342724@N04/36729615770/ (“https://creativecommons.org/licenses/by/2.0/” CC BY 2.0); L. Chesser, Apple_Watch_user_(Unsplash) (“https://commons.wikimedia.org/wiki/File:Apple_Watch_user_(Unsplash).jpg”) “https://creativecommons.org/publicdomain/zero/1.0/” CC0 1.0); Peter H. Charlton, Webcam on computer screen (“https://commons.wikimedia.org/wiki/File:Webcam_on_computer_screen.jpg”) (“https://creativecommons.org/licenses/by/4.0/deed.en” CC BY 4.0); (centre) P-H. Chan et al. (243) https://doi.org/10.1161/JAHA.116.003428 (Creative Commons Licence).

Photoplethysmography-based devices could provide a more convenient approach to assess vascular age. Photoplethysmography is an optical technique that captures the pulsatile change in vascular blood volume with each heartbeat. It is widely used in physiological monitoring, from its use in pulse oximeters for oxygen saturation assessment (7), to its use in toe blood pressure measurement for vascular assessment (8), and its use in smartwatches for heart rate monitoring (9). It has also been investigated as a modality with which to assess vascular age, although it is not widely used for this purpose. The photoplethysmogram (PPG) signal is influenced in two ways by vascular aging. First, the time taken for the PPG pulse wave to arrive at peripheral sites reduces with chronological age, since arterial stiffness and therefore pulse wave velocity (PWV) increase with chronological age, particularly in the central arteries such as the aorta (10). Second, the shape of the PPG pulse wave changes with chronological age (11) because it is influenced by both the speed of pulse wave propagation (12) and changes in the compliance of smaller, peripheral arteries that affect wave reflection (13). Indeed, some PPG-derived parameters have been found to correlate with age (14), providing insights into the effects of age on the vasculature. The PPG is already measured by many devices with a range of designs and potential applications (see Fig. 1): PPG-based devices come in a range of form factors (e.g., from fitness bands to earbuds); measurements can be made either in contact with the skin or remotely (e.g., by finger probe or by webcam); PPG-based devices are used in clinical settings (e.g., pulse oximeters) and in daily life (e.g., smartwatches); devices can be used for continuous measurements (e.g., wearables) and intermittent measurements (e.g., placing a finger on a smartphone’s camera); and measurements can be taken at different body sites (e.g., finger, wrist, and ear), and even simultaneously at multiple sites. Consequently, the PPG is an attractive and convenient modality with which to potentially assess vascular age.

This review summarizes the state-of-the-art on assessing vascular age from the PPG. It details the technical aspects of using the PPG to assess vascular age (with sufficient detail for engineers to develop the technology further), and translational aspects (aimed at clinicians and researchers). The following topics are addressed herein: indicators of vascular age which have been assessed from the PPG (see *What Indicators of Vascular Age Have Been Assessed?*, for all readers); methods used to derive these indicators of vascular age (see *How Have Indicators of Vascular Age Been Derived?*, primarily for engineers); methods used to assess their performance (see *How Has the Performance of PPG-Derived Parameters of Vascular Age Been Assessed?*, primarily for researchers); the performance of PPG-derived parameters in comparison to reference indicators (see *How Well Do PPG-Derived Parameters of Vascular Age Perform in Comparison to Reference Indicators?*, primarily for clinicians and researchers); their repeatability and reproducibility (in *How Repeatable and Reproducible Are PPG-Derived Parameters of Vascular Age?*, primarily for clinicians and researchers); their clinical utility (in *What is the Potential Clinical Utility of PPG-Derived Parameters of Vascular Age?*, primarily for clinicians and researchers); and resources and directions for future research (in *What Resources Are Available to Researchers?* and in *Future Research Directions*, primarily for researchers). Key messages for all readers are provided at the start of each section.

## METHODS

The following methods were used to conduct this “systematic search and review,” using a comprehensive search process to address broad research questions (15).

### Research Questions

The review was designed to address the following questions:

- What indicators of vascular age have been assessed from the PPG?
- How have indicators of vascular age been derived from the PPG?
- How has the performance of PPG-derived parameters of vascular age been assessed?
- How well do PPG-derived parameters of vascular age perform in comparison to reference indicators?
- How repeatable and reproducible are PPG-derived parameters of vascular age?
- What is the potential clinical utility of PPG-derived parameters of vascular age?
- What resources are available to researchers in this field?

### Search Strategy

Potential publications were identified in two steps. First, a manual search was conducted and the results were used to design a systematic search strategy. Second, this systematic search was conducted. The manual search returned 31 articles, whose titles were mostly found to include words from two themes: *1*) the PPG signal and *2*) vascular aging. Therefore, the systematic search was designed to identify publications with at least one search term corresponding to each theme in their title. The search terms are listed in Table 1. The following five search engines were used for the systematic search: ACM Digital Library, IEEE Xplore, PubMed, Scopus, and Web of Science. Electronic searches were performed on 9 June 2020 by P.H.C. No date range was used, ensuring that no restriction was placed on the date of publication. All publications identified in either the manual search or the systematic search were screened for inclusion. Further details of the search methodology are provided in appendix.

**Table 1.** Review methodology

The Search Strategy Used to Identify Potential Publications from Five Search Engines	
Search Theme	Search Terms
PPG signal	photoplethysmogra* (*additional characters), PPG, pulse contour, volume pulse, volume wave
vascular aging	age, aging, aging, BP, decomposition analysis, elasticity, hypertension, intensity analysis, PAT, PDA, peripheral, PWV, pressure, PTT, pulse arrival time, pulse transit time, pulse wave velocity, stiffness, time difference

Inclusion Criteria for the Review	
Criterion	Inclusion
Method	used ≥1 PPG signal
Indicator of vascular age	one or more of: *1*) arterial stiffness; *2*) blood pressure; *3*) endothelial function; *4*) intimal thickening; *5*) atherosclerosis; *6*) calcification; *7*) chronological age
Language	English
Participants	Human
Type of publication	Journal article
Source type	a primary report of performance, clinical utility, repeatability, or reproducibility.
Text availability	full text available

BP, blood pressure; PAT, pulse arrival time; PDA, pulse decomposition analysis; PPG, photoplethysmogram; PTT, pulse transit time; PWV, pulse wave velocity.

### Study Selection

Publications were screened against the inclusion criteria in Table 1 using the Rayyan web application (16). Briefly, to be eligible, publications had to report a method using at least one PPG signal to assess an indicator of vascular age. Indicators of vascular age were defined using the “functional and structural” biomarkers reported by Hamczyk et al. (1), with the addition of chronological age. The full list of indicators is provided in Table 1. Blood pressure (BP) was included as it “increases during aging and is associated with cardiovascular events and mortality” (1). Chronological age was included as it has been used as a surrogate indicator of vascular age. Screening was performed using abstracts and full texts. Conference abstracts were not included as they typically did not provide enough information to address the research questions. Screening was performed collectively by the authors.

## RESULTS AND DISCUSSION

### Source of Evidence

*Key messages:* 162 articles were included in the review, the majority of which were published since 2016.

**Figure 2.**
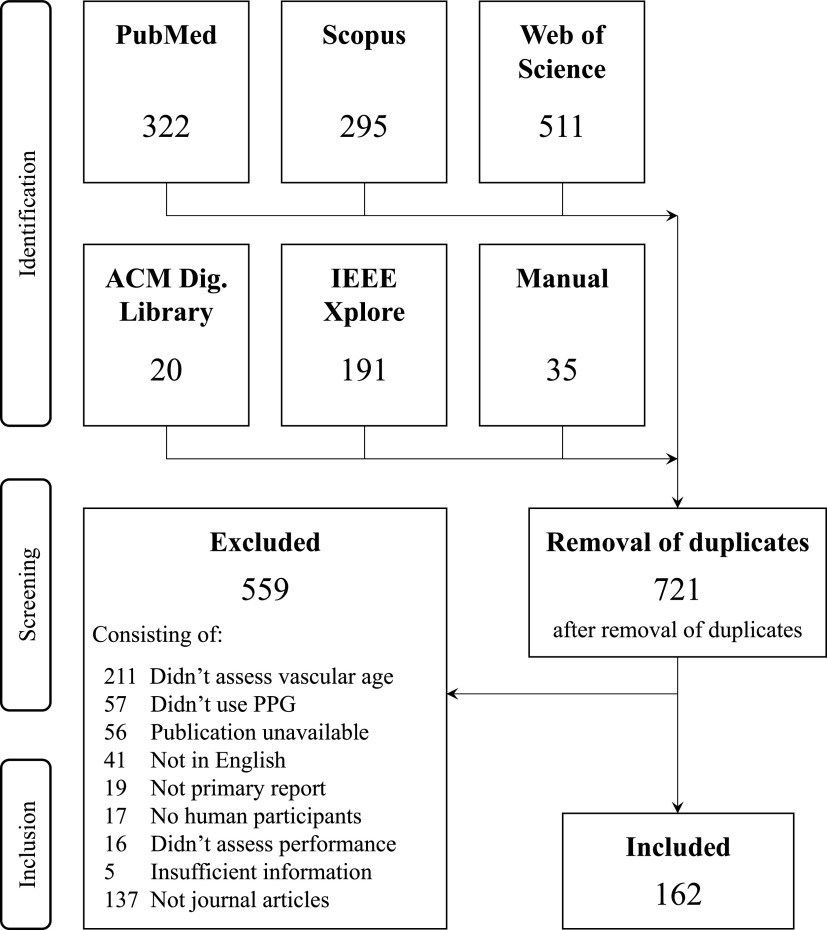
A summary of the identification and screening processes. PPG, photoplethysmogram.

**Figure A1.**
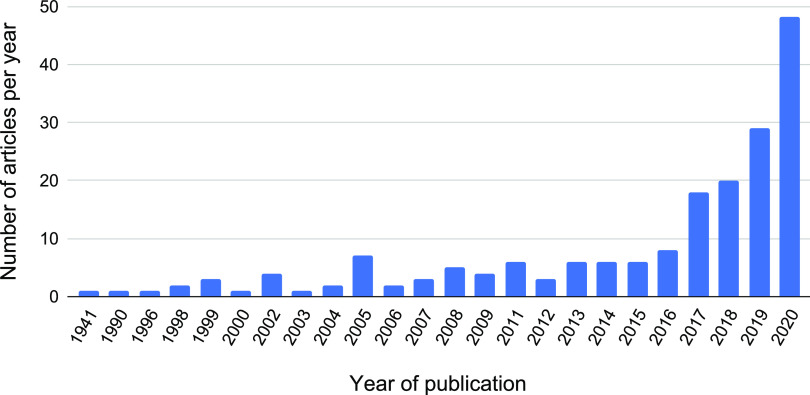
The number of included articles published per year.

A flow diagram is provided in Fig. 2 showing how publications were identified and screened for inclusion. A total of 1,372 publications were identified in the search. After removing duplicates, 721 publications remained. Screening excluded 559 publications leaving 162 articles for analysis (14, 17–177). Figure A1 (see appendix) presents the distribution of articles according to publication year. Most articles (60%) were published in the last five years, i.e., since 2016. Four journals accounted for almost a quarter of the articles: 19 (12%) in *Physiological Measurement*, 7 (4.4%) in *IEEE Transactions on Biomedical Engineering*, and 6 (3.8%) in each of *Sensors* and *American Journal of Hypertension*.

### What Indicators of Vascular Age Have Been Assessed?

#### Key messages.

The review identified three indicators of vascular age that have been assessed from the PPG: arterial stiffness, BP, and atherosclerosis. With increasing chronological age, arterial stiffness increases, BP rises, and atherosclerosis becomes more prevalent. Each of these impacts the arrival time of the PPG pulse wave at distal sites, and the shape of the pulse wave. Associations between PPG-derived parameters and chronological age have also been investigated. Although chronological age may be suitable for the development of techniques, it may not be suitable for their validation as it cannot distinguish between subjects of the same chronological age with different vascular ages.

The vast majority of articles focused on assessing BP, with fewer assessing arterial stiffness, and very few assessing atherosclerosis (see Table A1 in appendix for the numerical results). Several articles assessed the utility (clinical utility, repeatability, or reproducibility) of PPG-derived parameters. No articles were found in which endothelial function, intimal thickening, or calcification were assessed. Some articles investigated correlations between PPG-derived parameters and chronological age, although chronological age cannot distinguish between subjects of the same chronological age with different vascular ages (see Ref. 6). Therefore, the remainder of this review focuses on the following indicators of vascular age: arterial stiffness, BP, and atherosclerosis. The clinical relevance of each indicator of vascular age, and their effects on the PPG, are now described.

#### Arterial stiffness.

Arterial stiffness is an independent cardiovascular risk factor and a predictor of all-cause mortality (5). Arterial stiffness increases greatly with chronological age (10), resulting in increased PWV [as PWV is linked to arterial stiffness by the Moens-Korteweg equation (178)]. Arterial stiffness impacts the PPG in two ways. First, at higher PWVs the pulse transit time (PTT) from central to distal vascular locations is shorter, so the PPG pulse wave arrives earlier at distal sites. Second, the shape of the PPG pulse wave is influenced by PWV, since it is formed from incident and reflected waves whose arrival times are in part determined by PWV. The greatest change in PWV occurs in the aorta, with aortic PWV almost doubling from ∼6 m/s in young adults to 10 m/s in elderly adults (10). Consequently, PPG-based methods for assessing arterial stiffness are often designed to include the aortic pathway in PWV measurements (86) or to obtain a measurement of pulse wave shape, which is related to aortic PWV (73).

PPG-based approaches for assessing arterial stiffness could be used in both clinical and consumer settings. In the clinical setting, PPG-based devices provide an alternative approach to assess PWV with potential benefits of requiring less training to use, and being less operator dependent than existing devices. When used in consumer devices, PPG-based assessment of arterial stiffness could be used to assess cardiovascular risk in daily life and identify individuals who may be at increased risk and should be offered further cardiovascular assessment.

#### Blood pressure.

Elevated blood pressure (BP) is a leading risk factor for disease and mortality (179). BP rises with chronological age (180), and the vascular changes that occur with chronological age are accelerated at elevated BPs (3). At low BPs, elastin bears much of the stress in the arterial wall, whereas as BP increases the load is taken up by progressively more collagen that is much stiffer than elastin. This results in increased arterial stiffness and therefore increased PWV. Chronic increases in BP can also result in increased wall stiffness and thickness (181). The age-related change in BP varies between central (i.e., aortic) and brachial (i.e., arm) sites: the increase in central systolic BP in normal vascular aging is much greater than the increase in brachial systolic BP (182). Consequently, central BP should be preferred to brachial BP as a reference indicator of vascular age against which to compare PPG-derived indicators of vascular age. Indeed, it may be feasible to estimate a central BP waveform from a peripheral PPG waveform (141), as transfer functions have been used to relate PPG to BP waveforms at the same site (183), and to relate BP waveforms at peripheral and central sites (184). However, it may be unrealistic to use such an approach if local vascular properties impact the required transfer function, such as microvascular properties that impact the PPG.

There is potentially great benefit to assessing BP from the PPG. BP assessments could be incorporated into wearable devices such as smartwatches and fitness trackers for unobtrusive monitoring in daily life. This could help identify hypertension, and could be used to monitor BP trends such as the nocturnal dip, which has prognostic value (17). Furthermore, in the clinical setting the PPG provides an alternative approach to measure BP in peripheral locations such as the toe, by deflating a proximal cuff and identifying the appearance and disappearance of distal pulses indicating systolic and diastolic BP, respectively.

#### Atherosclerosis and PAD.

Atherosclerosis is a disease of the intima of the arteries, triggered by endothelial dysfunction. In more advanced cases, plaques form resulting in narrowing (arterial stenosis) of the arteries. This functional and structural pathophysiological process is a feature of vascular aging enabling its use as an indicator of vascular age (1). Atherosclerosis can reduce circulatory capability and cause end organ damage (185).

Atherosclerosis can impact the PPG in two ways. First, it has been found to be associated with increased arterial stiffness (186), with several possible mechanisms proposed linking atherosclerosis and arterial stiffness (187). Thus, atherosclerosis can result in similar changes to PPG PTT and shape as observed with increased arterial stiffness (see *Arterial stiffness*). Second, atherosclerosis can manifest in the lower limbs as PAD (155). PAD occurs when arteries carrying blood to the limbs narrow, often due to the build-up of plaque, causing a reduction in blood flow to the limbs (most often the legs). It shares common risk factors with coronary artery disease and stroke and its prevalence increases with age (188), rising from the fourth and fifth decade of life to ∼15% at age 70 and over (188).

It is important to identify PAD as it is associated with increased morbidity and mortality, and yet is straightforward to treat (189). However, PAD is under-recognized and under-treated (190). PAD is typically identified through the ankle-brachial index (ABI), the ratio of systolic BP at the ankle to that at the brachial artery, with ABI ≤ 0.90 indicative of PAD (191). PPG-based approaches for identifying PAD could potentially be automated and provide user-independent identification of PAD (23), in some cases identifying differences in PPG pulse wave shapes between limbs (106) as PAD can affect arterial function in each limb differently. Such approaches may be particularly useful for identifying PAD in primary care, with the advantages of being noninvasive and requiring minimal training (106). Although studies have demonstrated the feasibility of identifying PAD from bilateral differences in PPG pulse waves (98), it may not always be possible to differentiate between PAD and increased arterial stiffness, as they can have similar effects on the PPG.

### How Have Indicators of Vascular Age Been Derived?

**Figure 3.**
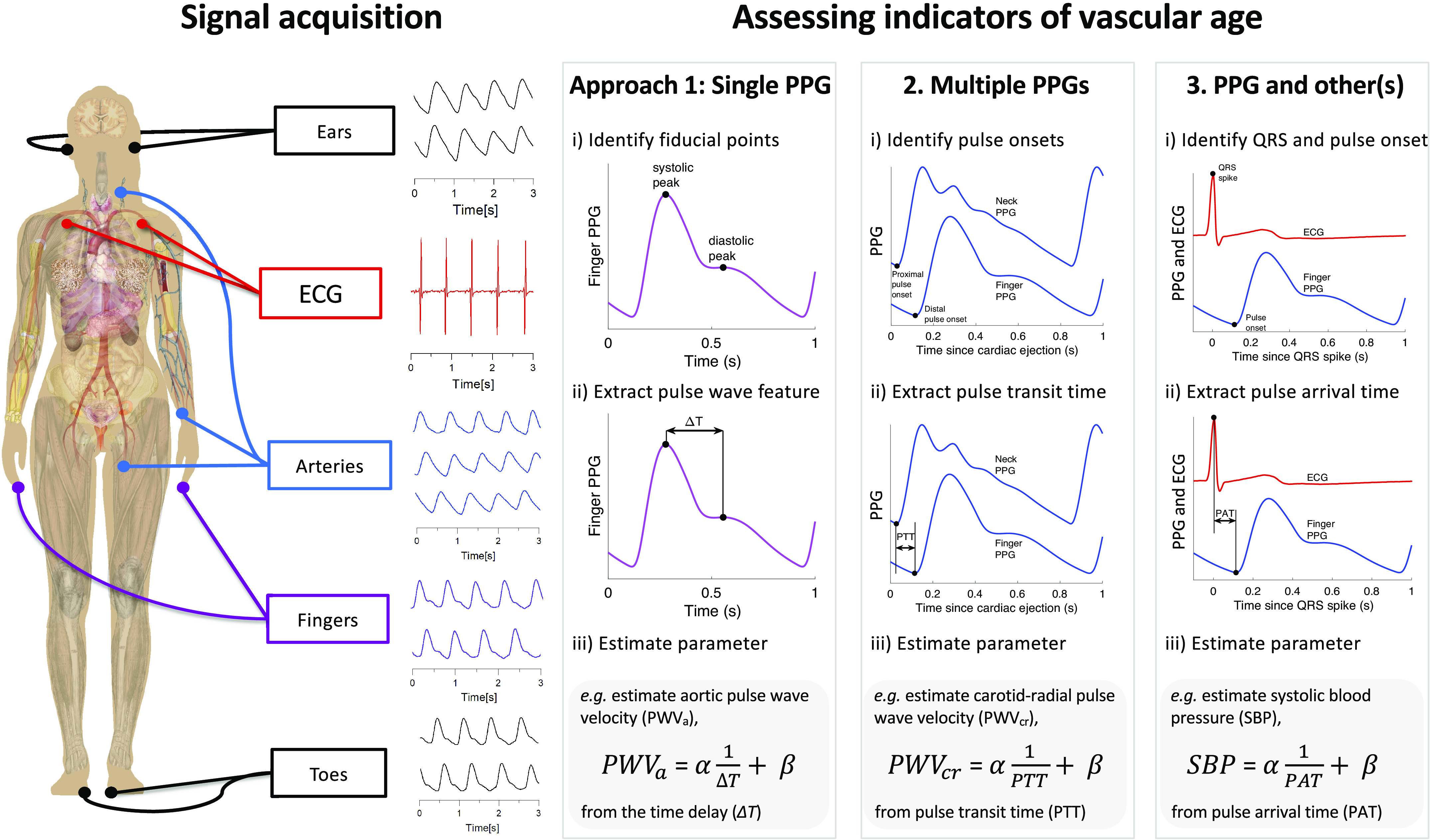
Three approaches for assessing indicators of vascular age from the photoplethysmogram (PPG): Signal(s) are acquired from single or multiple sites (*left*). One of three approaches is then used to derive a parameter of vascular age from the following signals: *1*) a single PPG, *2*) multiple PPGs, or *3*) PPG and other(s). An example of a regression equation for assessing an indicator of vascular age is provided for each approach: *i*) estimating aortic pulse wave velocity from the time delay between systolic and diastolic peaks on a PPG pulse wave; *ii*) estimating carotid-radial pulse wave velocity from the pulse transit time (PTT) between PPG pulse waves measured at different sites; *iii*) estimating systolic blood pressure from the pulse arrival time (PAT) between the QRS spike of an ECG signal, and the arrival of a PPG pulse wave at the finger. ECG, electrocardiogram; α and β, linear regression coefficients obtained during a calibration procedure. Sources: Mikael Häggström, Female shadow anatomy without labels (“https://commons.wikimedia.org/wiki/File:Female_shadow_anatomy_without_labels.png”) (public domain); “signal acquisition” signals—Institute of Biophysics, University of Belgrade; remaining PPG signals—the Pulse Wave Database under ODC PDDL v.1.0 (https://opendatacommons.org/licenses/pddl/1-0/) (4).

*Key messages:* Approaches to assess indicators of vascular age fall into three categories, as illustrated in Fig. 3: those which use a single PPG signal (based on pulse wave analysis), those which use multiple PPG signals (e.g., pulse transit time measurement between two PPGs), and those which use PPG and other signals (e.g., pulse arrival time measurement between the ECG and a PPG). Having used one of these approaches to derive a parameter from the PPG, a mathematical model is then often used to transform the parameter into an indicator of vascular age (such as converting pulse transit time to systolic blood pressure).

The number of articles that used each approach is now described, with results presented in Table A1. The most common approach was to use a single PPG signal (135 articles, 83%), which can be used with all PPG-based devices, including consumer devices (e.g., wristbands, smartwatches, and smartphones) and pulse oximeters. The next most commonly used approach was “PPG and other signals” (59 articles, 36%), which can be used with some advanced consumer devices [e.g., smartwatches that acquire both PPG and electrocardiogram (ECG) signals], and specialist clinical devices. The “Multiple PPG signals” approach was used least frequently (33 articles, 20%). Currently, it can only be used with specialist clinical devices.

The methods used to derive parameters from the PPG with each approach are now described, followed by a summary of how models have been used to transform PPG-derived parameters into indicators of vascular age.

#### Deriving parameters from a single PPG signal.

*Key messages*: Many methods have been proposed to derive parameters of vascular age from the PPG pulse wave, based on pulse wave analysis. These exploit the changes in pulse wave shape that occur in vascular aging. It is not yet clear which method is most suitable for assessment of vascular age.

Methods used to derive parameters of vascular age from a single PPG signal are based on pulse wave analysis—analysis of the shape of the pulse wave (192). Pulse wave analysis is perhaps most commonly used for the analysis of arterial BP signals, providing measures of pulse wave shape such as the augmentation index from applanation tonometry signals (193). It is already routinely used in cardiac output monitors to estimate cardiac output from the BP signal (194). The methods identified in this review to analyze a single PPG signal are summarized in Table 2.

**Table 2.** Methods used to derive parameters of vascular age from a single PPG signal (x)

**Pulse wave features**
• Time delay: between systolic (sys) and diastolic (dia) peaks on the pulse wave (Δ*T* in Fig. 5B) (167).
• Stiffness index (SI): a subject’s height divided by the time between sys and dia (Δ*T* in Fig. 5B) (73).
• Crest time (CT, also known as pulse risetime): the time from pulse onset (onset) to sys (103) (see Fig. 5B).
• Peak-to-onset time, corrected (P2Ocd): P2Ocd is the time interval between the sys and the following onset, divided by the pulse wave duration (140).
• Other time periods: including from: *1*) onset to dicrotic notch (dic) (20); *2*) sys to diastolic rise (176); *3*) sys to pulse end (176); *4*) dic to pulse end (20); *5*) diastolic rise to pulse end (176).
• Reflection index (RI): the ratio of dia and sys amplitudes (see Fig. 5B) (167).
• Augmentation index (AIx): the ratio of the amplitudes of *p2* and *p1*, defined as [*x*(*p*2) – *x*(onset)]/[*x*(*p*1) – *x*(onset)] (88, 135).
• Dicrotic notch: the presence or absence of dic (129) (see dic in Fig. 5).
• Dicrotic notch amplitude: (79) (see dic in Fig. 5).
• Class of PPG waveform: class as determined by pulse amplitude and dic positioning (57).
• Other amplitude features: e.g., widths of individual Gaussians obtained through pulse decomposition (17).
• Statistical measures “to quantify entropy, irregularity and frequency content” of a short period of PPG (e.g., 5 s) (17, 195).
• Standardized moments of pulse wave data: skewness to quantify asymmetry, and kurtosis to quantify outliers (69).
• Shape index: the area under the pulse wave falling outside the range of healthy pulse wave shapes (98).
• Areas under the pulse wave: *1*) under the whole pulse wave (132); *2*) from onset to the maximum upslope (ms) (176); *3*) from ms to sys; *4*) from onset to sys (109); *5*) from sys to diastolic rise (176); *6*) from diastolic rise to pulse end; *7*) ratio of systolic to diastolic areas (segmented at dic in Fig. 5) (151).
• Pulse widths calculated at the height of: *1*) half the pulse wave amplitude (151); *2*) other quantiles, from 10% to 75% of the pulse wave amplitude (50, 175). Pulse widths can be divided into the width before and after sys (50).
• Compliance index: the area under the pulse wave divided by the pulse pressure (119).
• Perfusion index (PI): the ratio between the amplitudes of pulsatile and nonpulsatile components of the infrared PPG signal (119).
• Pulse amplitude (AMP): the absolute pulse amplitude, *x*(sys) – *x*(onset) (57), calculated from a PPG waveform which has not been normalized.
• Modified normalized pulse volume (mNPV): defined as [*x*(*sys*) – *x*(onset)]/*x*(sys) (66), calculated from a PPG waveform which has not been normalized and retains its original offset.
**First derivative features**
• Slope of the rising front: the amplitude of ms, normalized by the pulse amplitude (133).
• Minimum rise time: the amplitude of the pulse wave divided by the amplitude of ms (96).
• Mean slopes: (i) between onset and sys; (ii) between sys and pulse end (151).
**Second derivative features**
• Fiducial point amplitudes: amplitudes of points on second derivative (*b*, *c*, *d*, and *e*), which are usually normalized by the amplitude of *a* (88) (see Fig. 5A).
• Aging index (AGI): defined as (*b* – *c* – *d* – *e*)/*a*, where *a*, *b*, *c*, *d*, and *e* are characteristic point amplitudes (88).
• Level-crossing features: the number of crossing of a contour line at a particular level on the second derivative, and the durations of the resulting segments (59).
**Combinations of features**
• Spring constant: defined as *x*′′ (sys)/[(*x*(*sys*) – *x*(ms)]/*x*(sys) (127), derived from a physical model of the elasticity of peripheral arteries.
• Combined IPAD index: the sum of: *1*) the area under the PPG pulse wave after dic divided by the area under the pulse wave before dic, and *2*) *d*/*a* (165).
• Minimum rise time (MRT): defined as [1/*x′*(ms)]·[*x*(*sys*) – *x*(onset)] (96).
• Time intervals of periods segmented according to the polarities of the first and second derivatives (162).
**Frequency domain analysis**
• Normalized power of harmonics (123).
• Frequency domain features (81, 196).
• Fast Fourier Transform analysis: Use of fast Fourier transform to extract amplitude and phase information from the PPG signal (150).
• Harmonic phase shift: the phase shift between the fundamental frequency and the first-harmonic (154).
• Instantaneous frequencies: extracted using the Hilbert–Huang transform (160).
• Frequency spectrum metrics: Summary measures of the frequency spectrum, including the amplitudes and frequencies of the highest peaks, energy, and entropy (41, 69).
• Spectral power in low (LF, 0.04–0.15 Hz) and high frequency (HF, 0.15–0.40 Hz) bands, and the LF/HF ratio (81).
• Very low frequency fluctuations: pulse amplitudes or baselines are low-pass filtered to leave fluctuations which occur over 30–80 beats (29).
**Features from multiple beats**
• Pulse rate variability parameters (17, 32).

PPG, photoplethysmogram.

**Figure 4.**
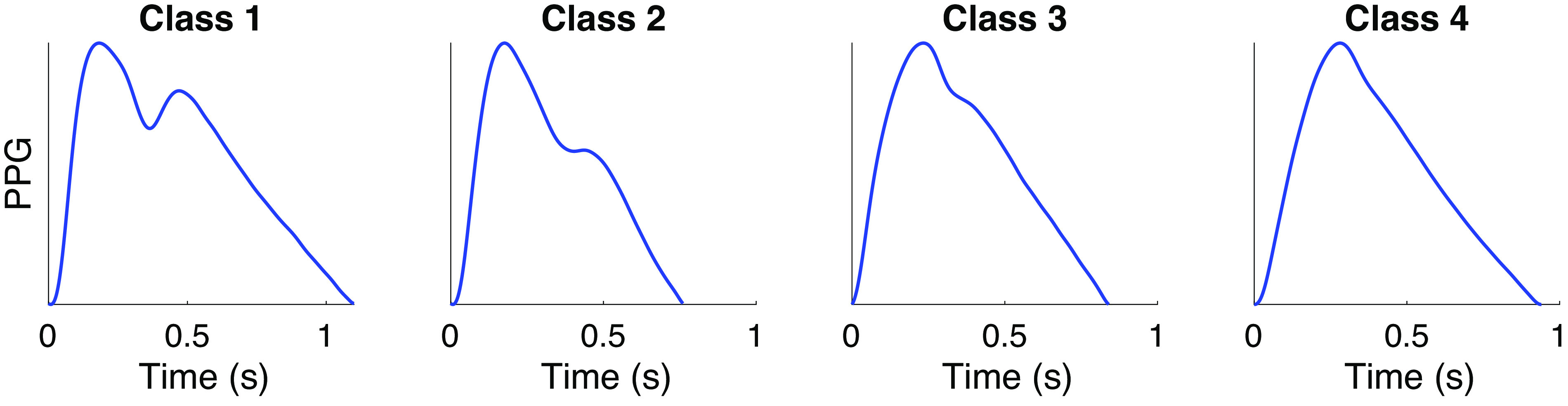
Classes of photoplethysmogram (PPG) pulse wave shape: Typical changes in PPG pulse wave shape with age, from young (*left*) to old (*right*). As described by Dawber et al. (244): *class 1* waves exhibit an incisura; *class 2* show a horizontal on the line of descent; *class 3* show a change in gradient on the downslope; *class 4* shows no evidence of a notch. Pulse waves were measured using infrared reflection mode photoplethysmography, and were obtained from the Vortal dataset (245). Source: P. H. Charlton, “Classes of photoplethysmogram (PPG) pulse wave shape (https://commons.wikimedia.org/wiki/File:Classes_of_photoplethysmogram_(PPG)_pulse_wave_shape.svg)” (CC BY 4.0).

Most of the methods quantify the shape of the PPG pulse wave, as it changes with chronological age (see Fig. 4). Pulse wave shape is influenced by both local factors [e.g., peripheral compliance (4, 167)] and systemic factors (e.g., large artery stiffness and cardiac ejection) (4, 88, 167). A dicrotic notch and a diastolic peak are visible on the downslope of *class 1* waves (see Fig. 4), which are commonly observed in young adults. These features diminish with chronological age, until they are typically no longer visible in elderly subjects (*class 4*). The pulse wave is composed of the incident wave from the heart and additional reflected waves. The speed of pulse wave propagation influences the timing of these waves, and therefore contributes to the dicrotic notch and diastolic peak characteristics (167). Consequently, vascular age has been commonly assessed from the shape of pulse waves using time- or frequency-domain (197) analysis.

**Figure 5.**
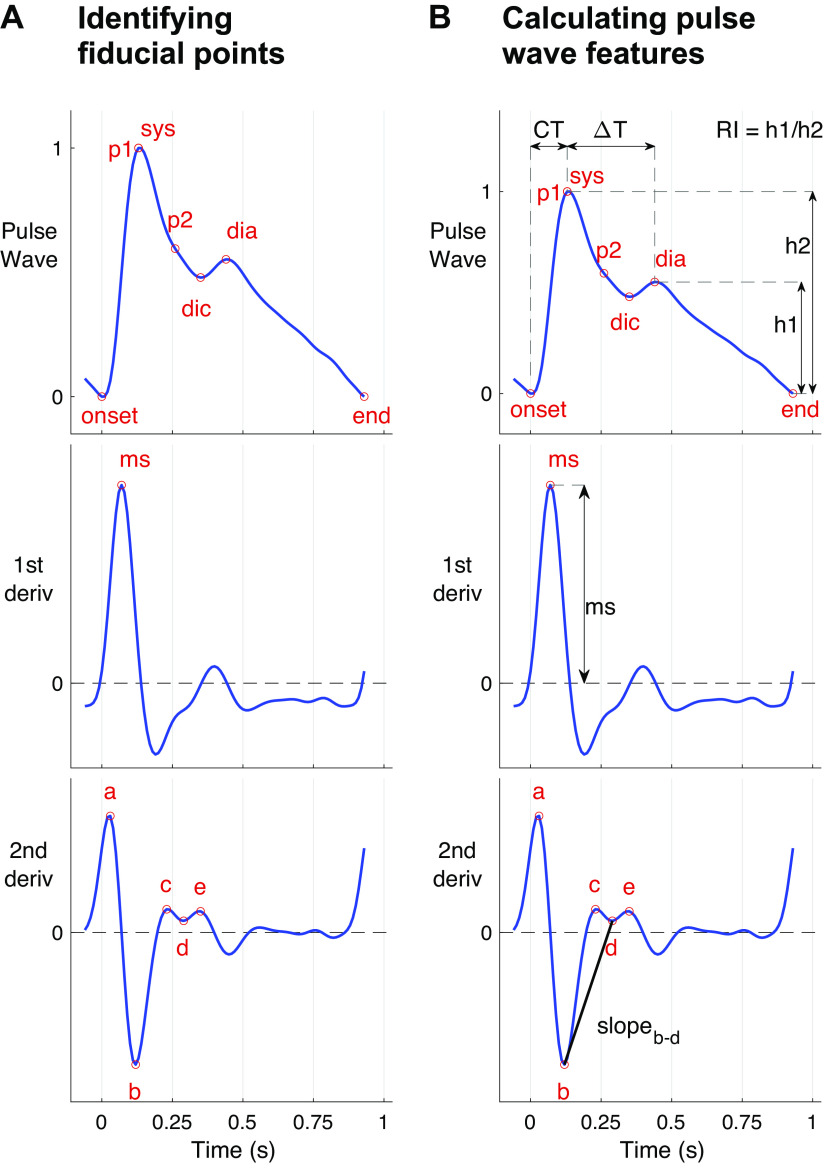
Extracting features from photoplethysmogram (PPG) pulse waves. Features can be extracted from a single PPG pulse wave in two steps: *A*) identifying fiducial points on the pulse wave, such as systolic (sys) and diastolic (dia) peaks, dicrotic notch (dic), early and late systolic peaks (*p1* and *p2*), the slope of the rising front (ms), and *a*, *c*, *e* peaks and *b* and *d* troughs of the 2nd derivative; and *B*) calculating features from the amplitudes and timings of these points, such as the time from pulse onset to sys (CT), the time from sys to dia (Δ*T*), the reflection index (RI), the maximum upslope (ms), and the slope between *b* and *d* troughs (slope*_b_*_-_*_d_*). Sources: *A*: P.H. Charlton, “Photoplethysmogram (PPG) pulse wave fiducial points” (https://commons.wikimedia.org/wiki/File:Photoplethysmogram_(PPG)_pulse_wave_fiducial_points.svg) (CC BY 4.0); *B*: P.H. Charlton, “Photoplethysmogram (PPG) pulse wave indices (https://commons.wikimedia.org/wiki/File:Photoplethysmogram_(PPG)_pulse_wave_indices.svg)” (CC BY 4.0).

Several parameters have been proposed to quantify wave shape, as illustrated in Fig. 5. When using time-domain analysis, these parameters are typically extracted by *1*) identifying fiducial points on PPG pulse waves and their derivatives (Fig. 5*A*) and *2*) calculating features (Fig. 5*B*) from the fiducial points. The features can be calculated from the timings and amplitudes of fiducial points (which can be normalized by pulse wave duration and amplitude), as well as slopes, areas, quadratic areas, and ratios of features (21, 69). Many features have been related to cardiovascular properties.

Several features have been extracted from the original pulse wave (Fig. 5*B*, *top*). The stiffness index (SI) and reflection index (RI) are commonly used, and both are influenced by the vascular state and cardiac ejection. The SI and RI are calculated from the timings and amplitudes, respectively, of the systolic (sys) and diastolic (dia) peaks on pulse waves (see Fig. 5*A*). These originate from an incident wave from the heart, followed by a temporarily spread reflected wave (assumed to consist of a number of reflected waves from around the circulation). The time delay between peaks (from which SI is calculated) is about four times the aortic pulse transit time (PTT), and is correlated with it (*r* = 0.75) (167). This relation is in line with reflections from the lower limbs (167). The RI (calculated from the relative amplitude of the peaks) has been found to be associated with acute changes in the stiffness of systemic arteries (167). Characteristics of the incident wave are also related to vascular aging (83), such as: the time of sys (CT), the slope of the rising front (ms), and a surrogate augmentation index (AIx). AIx is related to arterial stiffness and wave reflections. It is typically derived from the ascending aortic pressure waveform and calculated as augmentation pressure (the pressure difference between the first and second systolic peaks, *p1* and *p2*) divided by pulse pressure (198). The surrogate AIx calculated from the PPG pulse wave significantly correlates with augmentation indices calculated from radial BP pulse waves (*r* = 0.77) (135) and central BP pulse waves (*r* = 0.78 and 0.86) (88, 135). Although there are differences in the AIx derived from PPG and BP pulse waves, it has been observed that augmentation is usually positive (i.e., *p2* > *p1*) on the PPG pulse wave when it is positive in the BP pulse wave, and vice-versa (135). Several methods have been used to locate *p1* and *p2* on the pulse wave, including using the second (135), third (Fig. 5*A*), and fourth derivatives (199).

Several features have been extracted from the second derivative of the pulse wave (third panel down on Fig. 5*B*) (88, 165). Five distinct peaks and troughs can be identified: *a*, *b*, *c*, *d*, and *e*. The amplitudes of *b* to *e*, normalized by that of *a*, are typically used as parameters of vascular age. The parameters *b/a*, *c/a*, and *d/a* primarily describe changes in the systolic part of the pulse wave, since points *a* to *d* occur in systole.

Each of these methods requires an algorithm to identify fiducial points, which requires careful design, particularly as pulse wave shape varies greatly with age (see Fig. 4). In the case of younger subjects, the diastolic wave peak can be detected using the second zero crossing of the first derivative of the PPG. However, pulse waves from older subjects often do not contain a distinct diastolic peak, in which case the wave location can be identified from derivatives (90, 137). The interested reader is referred to Ref. 69 for details of dicrotic notch detection (dic in Fig. 5), and Ref. 200 for details of pulse decomposition methods to identify systolic and diastolic pulse waves. Differentiation amplifies higher frequency components of a signal, so noise should be filtered out before differentiation (90). It should be noted that arterial stenosis can result in much weaker or even complete disappearance of PPG pulses (201), affecting analyses of pulse wave shape.

Frequency domain analysis has also been used to extract features from the PPG pulse wave. The fast Fourier transform is used to describe the pulse wave, with most information contained in approximately the first 10 harmonic components (or below ≈10 Hz) (123). The fundamental frequency component corresponds to the heart rate, with higher frequency harmonics at multiples of the fundamental frequency. The magnitudes of the higher frequency components are typically normalized by the magnitude of the fundamental frequency component. The magnitudes of the normalized frequency components have been found to decrease in vascular aging (96, 123).

It is not yet clear which measures of pulse wave shape provide the best assessment of vascular age. Key technical considerations include: the physiological determinants of each feature (see Ref. 4 for examples); the reliability of pulse wave analysis algorithms for extracting features, particularly in older subjects; the site used for PPG measurement [since the pulse wave shape differs with measurement site (202)]. It would be beneficial to conduct a systematic study of different features of pulse wave shape, assessing their associations with reference indicators of vascular age, and assessing their clinical utility.

#### Deriving parameters from multiple PPG signals.

*Key messages:* Methods to assess vascular age from multiple PPG signals include *1*) measuring PTT, from which PWV can be calculated and *2*) comparison of pulse wave features between contralateral (opposite) limbs to identify PAD.

The methods used to derive parameters of vascular age from multiple PPG signals are summarized in Table 3. Most methods use two or more of the PPG measurement sites shown in Fig. 3 to acquire multiple PPG signals.

**Table 3.** Methods used to derive parameters of vascular age from multiple PPG signals

**Pulse transit time (PTT) and Pulse wave velocity (PWV)**
• Multisite PTT: the delay between PPG pulse waves measured at two sites, e.g., carotid-radial, carotid-femoral, femoral-ankle (89), ear-finger, ear-toe, finger-toe (24, 87, 94).
• Single-site, dual-sensor PTT: the delay between PPG pulse waves measured using two sensors a short distance apart [e.g., proximal and distal locations along the carotid artery (170)].
• Single-site, single-sensor PTT: the delay between PPG pulse waves obtained using different wavelengths of light at a single site, e.g., the delay between infrared and blue PPGs is indicative of arteriolar PTT (the time taken for pulse waves to propagate from the arteries to the capillaries) since the infrared and blue PPGs are indicative of the arterial and capillary pulses, respectively (53).
• Estimating a parameter from PTT and pulse wave features: PTT measured between PPG signals at multiple sites, and pulse wave features, were used as inputs to a model to estimate blood pressure (61).
• PWV: calculated from PTT and the arterial path length between measurement sites (PWV = path length / PTT).
**Multisite assessment of pulse wave features**
• Crest time (CT, a.k.a pulse risetime) is assessed at a toe on each foot. Peripheral arterial disease is identified if the CT at either toe exceeds a threshold (62).
**Multisite comparison of pulse waves**
• Multisite pulse wave feature comparison: bilateral comparison of PPG pulse wave features (such as timing, amplitude or shape characteristics) between limbs (106).
• Multisite pulse wave feature comparison under hyperemia: bilateral comparison of pulse wave features (such as amplitude) between limbs: one limb exposed to hyperemia through prolonged pressure cuff inflation, and the other acting as a control (118).
• Bilateral differences: assessing bilateral blood pressure differences between index fingers to assess risk of arteriosclerosis (159).

PPG, photoplethysmogram.

Methods using multiple PPG signals mostly assess PTT, the time delay between PPG pulse waves at two sites. PTT can be derived from two PPG signals measured from *1*) two distinct sites (e.g., finger and toe sites); *2*) two positions along an artery (e.g., lower and upper neck providing two measurements along the carotid artery); or *3*) a single site using two different wavelengths of PPG, which penetrate to different depths (indicating the time delay between pulse waves at different levels of the vasculature). There is a dichotomy between using distal sites for PTT measurement, which result in a longer PTT making it easier to measure differences in PTT (203), and using more central sites to ensure the PTT is more strongly influenced by the aorta (204). PTT can be used directly to assess vascular age, with lower values indicating a higher vascular age (87). It can also be used in conjunction with an arterial path length measurement to estimate PWV (94), allowing comparison with reference values and between subjects (10). Several body sites have been considered for PPG-based PWV measurements, including: carotid-radial, carotid-femoral, and femoral-ankle paths (89). Different algorithms have been used to extract the timing of pulse waves, and can have a large influence on the results. For instance, the use of the “maximum of second derivative” algorithm has been found to produce finger-toe PTT measurements that correlate most strongly with carotid-femoral PTT measurements (161). This algorithm uses the *a* point on the second derivative as the marker of pulse wave timing, shown in Fig. 5*A*.

Multisite photoplethysmography (MPPG) can be used in a number of ways to detect PAD. With the expectation of bilateral similarity in PPG features for healthy subjects (similarity between opposite limbs) (205), MPPG can measure the relative delays in pulse arrival time (and/or features of normalized pulse shape) between contralateral body sides (e.g., between the great toes). Significant differences can indicate the likely presence of PAD (106), as PAD is often asymmetric in nature (due to differing locations and severities of atherosclerosis). MPPG technology can also speed up assessments by studying multiple peripheral sites simultaneously, rather than taking measurements sequentially at each site. This has the benefit that PAD can be detected even if only the pulse wave features at one limb meet the criteria for diagnosis.

MPPG has also been used to assess PWVs at several sites across the body, including the ears, fingers, and toes. However, care is needed when measuring the PWV as it can be inaccurate when significant PAD (or plaque) is present in a limb. For instance, arterial stenosis of the leg has been found to increase PTT through the leg by 20–80 ms (201), which is comparable with normal PTT measurements (such as finger-toe PTTs of 30–100 ms). If the PAD is isolated to one limb then the contralateral side could be used instead for PWV measurement (206).

#### Deriving parameters from the PPG and other signals.

*Key messages:* Methods to assess vascular age from a PPG signal and another signal include *1*) measuring pulse arrival time (PAT), *2*) measuring PTT, and *3*) using a PPG sensor and a pressure cuff to assess BP.

These methods are detailed in Table 4, and now described in turn.

**Table 4.** Methods used to derive parameters of vascular age from a PPG signal and another simultaneous signal

**Pulse arrival time (PAT)**
• PAT: calculated as the delay between the R-wave in the electrocardiogram (ECG) signal and arrival of a peripheral PPG pulse wave (207).
• Segmental PAT: the difference between PAT values at different body sites such as finger and ear, or toe and ear (24).
• PAT variability: beat-to-beat PAT variability (23).
**Pulse transit time (PTT)**
• PTT: the delay between two pulse waves, typically one indicating ejection from the heart (e.g., using impedance cardiography), and a PPG pulse wave measured peripherally (207).
• PTT calculated from PAT and pre-ejection period (PEP): the difference between PAT and PEP, i.e., PTT = PAT - PEP (120).
**Pulse wave velocity (PWV)**
• PAT-derived PWV: a surrogate for PWV, calculated from PAT and a measure of arterial path length (112).
Using PPG and blood pressure (BP) measurements to assess peripheral compliance
• Peripheral compliance index: the ratio of PPG pulse amplitude to BP pulse amplitude (at finger (145) or arm (119)).
**Using PPG and other signals to measure BP and ankle-brachial index (ABI)**
• Volume-clamp BP measurement: A servo-controlled, inflatable finger cuff maintains a constant arterial diameter by continuously adjusting its pressure to be equal to the arterial pressure, based on a PPG measurement (156, 208).
• Identifying SBP using proximal cuff deflation: A pressure cuff is placed upstream of the PPG measurement site (arm (97), ankle (95) or toe (93, 124)), and inflated above SBP. The reappearance of a PPG pulse wave upon deflation indicates SBP. A second PPG measurement on the opposite limb can be used to reduce noise (111).
• Ankle-brachial index (ABI): systolic BP (SBP) at the ankle (identified using proximal cuff deflation) divided by SBP at the arm (measured using a sphygmomanometer) (95).

PTT can be measured from the time delay between ventricular ejection and subsequent arrival of a PPG pulse wave at distal location. The time of ventricular ejection (corresponding to the pulse wave leaving the heart) can be obtained from ballistocardiography (65), seismocardiography (30), continuous wave radar (42), phonocardiography (68), or impedance cardiography (120) signals, or noncontact signals obtained using imaging PPG (164) or microwave sensors (209).

PAT is the time delay between the R-wave of the ECG and PPG pulse wave arrival, which includes not only the PTT from the heart to the PPG measurement site, but also the pre-ejection period (PEP, the time between ventricular depolarization and ventricular ejection). Consequently, while PAT decreases with chronological age due to its relationship with PTT (12), it cannot be considered a direct surrogate for PTT (210). Changes in segmental PAT (the difference between PATs at different sites) may be indicative of changes in arterial distensibility (102). PAT can be used to derive a surrogate for PWV, by using a measure of the path length along which the pulse wave travels [which can be estimated from height (114)] (91).

PTT can be estimated by measuring PEP and PAT separately, and then subtracting PEP from PAT (42, 120). This approach can be used to obtain PTT measurements when it is difficult to measure PTT directly. It accounts for inter- and intra-subject variability in PEP, providing potential improvement over using PAT alone. PEP can be estimated as the time delay between the R-wave of the ECG and a time of aortic valve opening obtained from any of: the *B* peak of an impedance cardiography signal (120); or the *I* peak of the ballistocardiogram signal (65); or the maxima of the seismocardiogram (30); or the *S1* sound in phonocardiogram (68); or by using noncontact sensors based on video or microwaves (211, 212). This approach allows PTT measurements to be obtained from devices in contact with a single point on the body [such as weighing scales (65) and wearable chest sensors (30, 68)], and from noncontact cameras (212). Consequently, the approach has potential advantages over devices that measure PTT from pulse waves at two locations, allowing measurements to be taken in daily life (e.g., scales or camera) and devices to be miniaturized (e.g., chest sensor).

The PPG can be used alongside a pressure cuff to assess several parameters. First, the peripheral compliance index, which decreases with chronological age (145), can be estimated from pulse pressure and PPG pulse wave amplitude (119, 145, 171). Second, the volume-clamp method can be used to measure BP continuously (156, 208). This approach is used by several commercially available devices (213). Third, systolic BP (SBP) can be identified upon deflating a cuff proximal to a PPG probe (97) [and can be combined with Korotkoff sound measurements to increase accuracy (80)]. Finally, the ABI can be calculated from a routine brachial SBP measurement, and an ankle SBP measurement obtained using a cuff and PPG probe (95, 113).

The use of a PPG signal and another signal has the advantage that the signals can often be acquired at a single site, and there are several potential sites and measurement devices. Potential sites include measuring signals at: the ear (30), face (209), neck (52), arm (97), wrist (52), finger (145), chest (68), ankle (95), foot (65), and toe (87). In addition, several types of device have been used, including: eye glasses (214), weighing scales (65), and video cameras (209, 212).

#### Using models to assess indicators of vascular age from PPG-derived parameters.

*Key messages:* Mathematical models can be used to transform PPG-derived parameters into indicators of vascular age. Several types of model have been used, including *1*) biophysical models, based on laws of cardiovascular mechanics; *2*) statistical models, such as regression analysis; and *3*) machine learning (ML) and deep learning (DL) models.

The models identified in this review are summarized in Table 5, and are described.

**Table 5.** Models used to assess indicators of vascular age from PPG-derived parameters

**Biophysical models**
• Biophysical models: mostly use the Moens–Korteweg or Hughes equations to relate the vessel wall elastic modulus to PWV and distending pressure, respectively (208), to estimate systolic (SBP), diastolic (DBP) and pulse pressure (PP) from PPG-derived parameters. Regression models are used to estimate BPs from PAT or PTT (163) (e.g., linear, logarithmic, inverse square, or inverse). • Models can account for additional factors: such as the pulsatile change in blood vessel diameter (estimated as PPG intensity ratio) (146), and the viscous effects of blood flow (55). • Windkessel modeling: use of a Windkessel model to assess arterial compliance (103).
**Statistical models**
• Auto-regressive models: (with exogenous input—ARX, and moving-average—ARMA) have been used to estimate the central BP waveform (141) and arterial SBP and DBP (174) from a PPG signal. • Estimating measures of vascular age: various regression models (e.g., linear, inverse, quadratic, exponential, partial least-squares) are used to estimate BPs from single PPG features (44, 59, 67, 79).
**Machine learning (ML) models: estimation**
• Estimating measures of vascular age from a single PPG pulse wave: the pulse wave, its first and second derivatives are used as inputs to a ML algorithm [e.g., nonlinear regression (39), deep neural network (41), support vector machine (SVM) (46)] to estimate numerical values (e.g., BPs). • Estimating measures of vascular age from pulse wave features: features are used as inputs to a ML algorithm [e.g., AdaBoost (66), random forest (35), artificial neural network (ANN) (150), regression tree (175)] to estimate numerical values (e.g., BPs). • Estimating measures of vascular age from multiple PPGs: features derived from multisite PPGs are used as inputs to ML algorithms (e.g., SVM) to estimate numerical values [e.g., SBP, DBP (61), and ABI (76)]. • Estimating measures of vascular age from PPG and other signals: PAT and other time and complexity features from the electrocardiogram and PPG, and PPG-derived features, are used as inputs to a ML algorithm (e.g., regularized linear regression, multiadaptive regression, back-propagation error neural network, convolutional neural network (CNN), SVM) to estimate numeric values, e.g., arterial DBP and SBP (31, 34, 45, 54, 67, 72). Associations with chronological age have also been assessed (47). • Estimating measures of vascular age from PPG and demographics: use of time-, frequency-domain and statistical features of PPGs along with demographic data as an input to a ML algorithm (e.g., ensemble trees, Gaussian process regression, multiple linear regression) to estimate numeric values, e.g., SBP and DBP (18, 69).
**Machine learning (ML) models: classification**
• Classifying pulse waves: use of a ML algorithm [e.g., K-nearest neighbor (KNN), CNN] to classify a pulse wave or a PPG signal transformation into a diagnostic category, e.g., normo-, prehyper- and hyper-tension (28, 74). • Classifying sets of pulse wave features: use of a ML algorithm [e.g., SVM, ANN, decision trees or KNN] to classify a set of pulse wave features into a diagnostic category, e.g., low or high PWV (105), normal or abnormal BP (56), normo-, prehyper- and hyper-tension (21, 75). • Classifying and then estimating measures of vascular age based on category: use of two-step ML algorithms to classify PPG features into BP categories (e.g., using KNN) and then estimate numeric values (e.g., SBP and DBP estimated using regression trees optimized for each BP category) (40).
**Machine learning (ML) models: miscellaneous**
• Extracting features and estimating measures of vascular age from single PPG: use of ML algorithm (e.g., CNN) to extract morphological features from a PPG segment (64) or its spectrogram (70) to estimate numerical values (e.g., BPs). • Improving the assessment of vascular aging: use of long short-term memory networks to capture temporal dependencies between PPG features (e.g., extracted by CNN) to better track changes in measures of vascular aging (e.g., BP) (17, 64, 77). • Reducing the feature vector: use of a ML algorithm (e.g., ANN) to nonlinearly map PPG features to reduce feature vector before estimating measures of vascular age (e.g., BPs) (50). • Reconstructing other signals: use of wavelet neural network (149) or auto-regressive model (141) to estimate BP waveform from PPG waveform.

PAT, pulse arrival time; PPG, photoplethysmogram; PTT, pulse transit time.

Biophysical models use laws of cardiovascular mechanics to model the relationship between a PPG-derived parameter (commonly PTT or PAT) and an indicator of vascular age (such as BP). Such models have the advantage of being based on known physiological relationships, such as the link between arterial stiffness and BP, or the Windkessel model of blood flow.

Statistical models, such as regression analysis, have been used to estimate BP from PPG-derived features. Different models may be required for different use cases. For instance, different models may be required to estimate BPs from different anatomical sites [e.g., finger and wrist (202)], and to estimate systolic or diastolic BPs (69). Statistical models have the advantage that the relationships encoded in them are learnt from data.

As in many other fields, machine learning and deep learning (ML and DL) models have also received much attention for assessing vascular age (40, 70). They have been mostly used to estimate BP, or to classify subjects into diagnostic categories such as normo-, prehyper-, and hypertension. ML and DL models have the advantage that not only can they take PPG-derived parameters as inputs, but they can also take the PPG pulse wave, its spectrum or its derivative, directly as an input (75), as well as demographic information (18, 69). This avoids the need for feature extraction. DL models can capture highly complex relationships observed in training data, but have the disadvantages that they can require substantial computing resources, and are often not interpretable. A challenge in the development of ML models is to avoid “overfitting”—the development of a model that is highly specific to the training data set, and not generalizable to external data sets. To address this, feature selection algorithms have been used to reduce an initial set of features to the most valuable ones, using algorithms such as the Relief feature selection algorithm (69, 176), analysis of relevance and redundancy (21), or nonlinear mapping.

An important aspect of model development is the manner in which data are used to train, validate, and independently test a model. Cross validation allows a single data set to be used for both model training and validation, which is convenient for initial development (18, 35, 66, 77). However, to obtain reliable results, data sets should ideally be divided into training, validation, and testing sets (70), and models should be tested on external data sets. Several articles have used subject-specific model training to improve performance (41, 64, 70), which may become increasingly feasible with the widespread use of PPG-based wearables, allowing a subject-specific model to be trained using an individual’s historical data.

### How Has the Performance of PPG-Derived Parameters of Vascular Age Been Assessed?

#### Key messages.

Studies of PPG-derived parameters of vascular age have mostly been conducted in healthy adults, with small sample sizes. Performance has been assessed against a range of reference indicators of vascular age, using several different statistical techniques.

The characteristics of the subjects in studies of PPG-derived parameters of vascular age are summarized in Table A2, and are described. Most studies included <100 subjects, indicative of proof-of-concept studies. Most studies included young and middle-aged adults. Few studies were conducted in children, who may well benefit from vascular age assessment (215). The sex of subjects was more frequently skewed toward males than females. Most studies included apparently healthy subjects, whereas few included subjects with diabetes or PAD. Few studies have been conducted on population cohorts, which will be important to investigate the potential utility of widespread vascular age assessment.

Key aspects of the experimental methodologies used to assess PPG-derived parameters of vascular age are summarized in Table A3, and are described. Some studies used gold standard reference indicators of vascular age (e.g., invasive BP and carotid-femoral PWV), whereas others used more readily available reference indicators (e.g., noninvasive BP and PWVs acquired along alternative arterial paths). Since most clinical evidence on using BP for decision making is based on brachial cuff measurements, it is still valuable to assess BP estimates against noninvasive BPs. Chronological age was also commonly used as a surrogate indicator of vascular age. PPG-derived parameters were compared with reference indicators using statistics indicative of: correlation, agreement, error, and classification ability. Correlation measures are helpful for the development of novel indices, while the limits of agreement technique is helpful for assessing agreement between estimated and reference parameters (216) [e.g., using grand means and standard deviations to weight each subject’s data equally (26)]. The performance of BP estimation techniques can be compared against the Association for the Advancement of Medical Instrumentation (AAMI) and the European Society of Hypertension’s (ESH) guidelines (217). Classification statistics (such as sensitivity, specificity, and *F*1-score) can be used to assess the ability of PPG-derived parameters to classify subjects into risk categories (such as hypertensive and normotensive). Most studies used a single data set, although some used multiple data sets, facilitating external validation (218). Studies using openly available data sets (see *What Resources are Available to Researchers?*) should report the subjects used in analyses to aid reproducibility.

### How Well Do PPG-Derived Parameters of Vascular Age Perform in Comparison to Reference Indicators?

#### Key messages.

Several larger studies have compared PPG-derived parameters to reference indicators of vascular age, including comparisons of with: carotid-femoral PWV; brachial BP; and the presence of PAD.

Selected larger studies (with >40 participants) comparing PPG-derived parameters of vascular age to reference indicators are presented in Table 6.

**Table 6.** Selected studies comparing PPG-derived parameters of vascular age to reference indicators

Study	PPG Parameter	Subjects (Dataset)	Reference Indicator	Performance
**Pulse wave velocity (PWV)**				
Tsai et al. (94)	Finger-toe PWV	100 healthy	carotid-femoral (cf) PWV	Finger-toe PWV correlated with cfPWV (*r* = 0.67, *P* < 0.01).
Millasseau et al. (73)	Stiffness index	87 healthy	cfPWV	Stiffness index correlated with cfPWV (*r* = 0.65, *P* < 0.0001).
von Wowern et al. (139)	Aging index (AGI)	112 pregnant and nonpregnant	cfPWV	Heart rate-adjusted AGI correlated with cfPWV (*r* = 0.64, *P* < 0.0001).
Wei (127)	Spring constant	70 diabetic	cfPWV	Spring constant correlated with cfPWV (*r* = –0.72, *P* < 0.001).
Jang et al. (140)	Corrected peak-to-onset time (P2Ocd)	123 healthy	brachial-ankle (ba) PWV	P2Ocd correlated with baPWV (*r* = –0.77 and *r* = –0.68 for male and female, *P* < 0.001). Mean absolute percentage error was 7.53 ± 5.37% for average values, and 3.22 ± 1.47% for each cardiac cycle.
**Pulse transit time (PTT)**				
Obeid et al. (161)	Finger-toe PTT, finger-toe PWV	101 healthy and hypertensive	cfPTT, cfPWV	Correlation coefficient, root-mean-square error, and mean ± SD error were 0.90 (*P* < 0.001), 5.3 ms, −10.6 ± 5.5 ms between finger-toe PTT and cfPTT; and 0.87 (*P* < 0.001), 0.7 m/s, and 0.3 ± 0.8 m/s between finger-toe PWV and cfPWV.
Alivon et al. (86)	Finger-toe PTT, finger-toe PWV	86 healthy, hypertensive, and cognitively impaired	cfPTT, cfPWV	Correlation coefficient and mean ± SD error were 0.77 (*P* < 0.0001) and –17.5 ± 19.7 ms between finger-toe PTT and cfPTT; and 0.66 (*P* < 0.0001) and 0.2 ± 2.5 m/s between finger-toe PWV and cfPWV.
**Systolic (SBP), diastolic (DBP) and mean (MBP) blood pressure (BP)**				
Nitzan et al. (87)	Finger-toe PTT, toe PAT	44 healthy	Brachial BP	Finger-toe PTT and toe PAT correlated with SBP (*r* = –0.52, *P* < 0.01, and *r* = –0.67, *P* < 0.0001), but not with DBP.
Xing et al. (35)	19 pulse wave and 2nd derivative features	1,249 healthy and hypertensive	Brachial BP	Correlation coefficient and mean ± SD error for subjects ≤ 50 yr were: 0.86 and 0.45 ± 11.3 mmHg for SBP, and 0.83 and 0.31 ± 8.55 mmHg for DBP; and for > 50 yr: 0.79 and –0.68 ± 14.1 mmHg for SBP, and 0.81 and –0.20 ± 9.0 mmHg for DBP using a random forest algorithm.
Hasanzadeh et al. (66)	Pulse wave features	942 critically ill (Cuffless BP Estimation)	Invasive BP	Correlation coefficient, mean ± SD error, and mean absolute error were 0.78, 0.09 ± 10.38 mmHg and 8.22 mmHg for SBP, 0.75, –0.02 ± 5.53 mmHg and 4.58 mmHg for MBP, and 0.72, 0.23 ± 4.22 mmHg and 4.17 mmHg for DBP estimation using an AdaBoost algorithm.
Khalid et al. (40)	Pulse area, rise time, width at 25% amplitude	282 critically ill (MIMIC) and anesthetized (University of Queensland)	Brachial BP	Mean ± SD error were 0.07 ± 7.1 mmHg for SBP, and –0.08 ± 6.0 mmHg for DBP estimation using BP category-specific regression tree algorithms.
**BP category**				
Liang et al. (28)	PPG scalogram	121 critically ill (MIMIC)	Invasive BP category	F1 scores for classification as normotensive (NT), prehypertensive (PHT), and hypertensive (HT) were 0.81 (NT vs. PHT), 0.93 (NT vs. HT), and 0.83 [(NT + PHT) vs. HT] using a convolutional neural network.
**Chronological age**				
Takazawa et al. (88)	AGI	600 healthy and arteriosclerotic	Chronological age	AGI increased with age (*r* = 0.80, *P* < 0.001).
Hashimoto et al. (14)	AGI, b/a, d/a	848 healthy and hypertensive	Chronological age	AGI, b/a and d/a correlated with age (*r* = 0.42, *r* = –0.35, and *r* = 0.37, respectively, with *P* < 0.001).
**Atherosclerosis category**				
Allen et al. (106)	Toe PPG shape index, toe PAT, pulse amplitude	111 healthy and peripheral artery disease (PAD)	Ankle-brachial index (ABI)	Accuracy (κ) of significant and higher-grade disease detection using: shape index 91% (0.80) and 90% (0.65); bilateral difference in PAT to pulse foot 86% (0.71) and 90% (0.71); bilateral difference in PAT to pulse peak 86% (0.70) and 92% (0.76); pulse amplitude 66% (0.20) and 81% (0.34).
Peltokangas et al. (27)	Amplitude ratios, AGI	82 healthy and atherosclerotic	Abnormal ABI	Area under the ROC curve was 0.70 and 0.79 for finger and toe AGI, respectively, and 0.79 for the best performing toe amplitude ratio.
Jönsson et al. (95)	PPG ABI	43 healthy and PAD	Doppler ABI	PPG ABI correlated with Doppler ABI (*r* = 0.89). Mean ± SD error was 0.05 ± 0.12.

PPG, photoplethysmogram.

Moderate correlations have been observed between reference PWVs (or PTTs) and those derived from PPG signals. For instance, coefficients with absolute values from 0.64 to 0.72 have been found between reference PWVs and PPG-derived parameters (finger-toe PWV and pulse wave parameters) (73, 86, 94, 127, 139). High correlations of 0.77 and 0.90 were found between reference PTTs and PPG-derived finger-toe PTT, with finger-toe PTT slightly overestimating carotid-femoral PTT by 10.6 and 17.5 ms (86, 161). Differences between PPG-based finger-toe measurements, and applanation tonometry-based carotid-femoral measurements, include: the finger-toe pathway including more peripheral vasculature; and the PPG pulse wave having a different morphology to pressure pulse waves, potentially impacting timing measurements. Moderate correlation coefficients of −0.52 and −0.67 were found between PPG-based finger-toe PTT as well as toe PAT and SBP (but not DBP) (87).

Studies have demonstrated the difficulty of estimating BP precisely from pulse wave features. BP estimates obtained from pulse wave features using ML algorithms can exhibit low bias (smaller than 0.68 mmHg), although achieving a low enough SD error of ≤8 mmHg (as required by AAMI standards) remains a challenge (35, 66). The required level of precision has been achieved by using a two-step algorithm in which pulse waves are categorized as hypo-, normo-, or hypertensive, and then BP is estimated using a model specifically for that category (40). Accurate classification into normo-, prehyper-, and hypertension BP categories has been achieved using PPG scalograms as inputs to a convolutional neural network (28). The performance of commercially available devices for continuous, noninvasive BP monitoring using the volume-clamp method was reported in a recent meta-analysis: substantial differences were found between BP estimates and reference invasive measurements, with population limits of agreement for SBP of −36 to 28 mmHg (213).

Varying strengths of correlation have been reported between indices of pulse wave shape and chronological age. The “aging index,” calculated from points on the second derivative of the pulse wave (as detailed in Table 2), has been found to be highly correlated with chronological age (*r* = 0.80) (88). A later study confirmed this relationship although the observed correlation was lower (*r* = 0.42) (14).

PPG parameters have been found to be useful in detecting atherosclerotic disease, particularly PAD. Parameters obtained at the toe, such as shape index and PAT, agreed well with ABI for significant and higher-grade PAD detection with classification accuracy above 86% (106). Toe amplitude ratios as well as the aging index from the finger and the toe can discriminate between normal and abnormal ABIs (27). Also, it has been proposed that PPG probes could replace conventional Doppler ultrasound probes in ABI measurement since the two methods correlated well (*r* = 0.89) and PPG-based ABI had only a small bias of 0.05 (95).

### How Repeatable and Reproducible Are PPG-Derived Parameters of Vascular Age?

#### Key messages.

PPG-derived PWVs have been found to have high repeatability and reasonable reproducibility, as have those parameters of pulse wave shape that are thought to be indicative of large artery stiffness.

The repeatability and reproducibility of PPG-derived parameters of vascular age are important aspects of their potential utility. Repeatability “refers to the variation in repeat measurements made on the same subject under identical conditions” (219), such as repeated measurements taken from a subject in a short period of time using the same device (usually within minutes while the subject is at rest). On the other hand, reproducibility “refers to the variation in measurements made on a subject under changing conditions” (219), such as measurements made by different device operators, or over an extended period of time such as days or weeks. A summary of studies reporting the repeatability or reproducibility of PPG-derived parameters of vascular age is presented in Table 7.

**Table 7.** Studies assessing the repeatability or reproducibility of PPG-derived parameters of vascular age

Study	PPG Parameter	Subjects	Delay	Findings
**Pulse wave velocity (PWV)**				
Loukogeorgakis et al. (89)	PWV along: carotid-femoral, arm and leg	Healthy 10 min: 10 3 h: 5	10 min 3 h	Coefficient of variation (CV): -carotid-femoral: 5.7% (10 min), 6.3% (3 h) -arm: 5.6% (10 min), 13.0% (3 h) -leg: 4.6% (10 min), 16.1% (3 h)
Tsai et al. (94)	Finger-toe PWV	20 healthy	20 min	Intra-class correlation coefficient (ICC): 0.959 Limits of agreement: 0.09 ± 0.69 m/s CV: 5.8%
Nabeel et al. (170)	Local carotid PWV	35 healthy	Beat-to-beat 10 s	CV: from 4.15% to 11.38% (beat-to-beat) Limits of agreement: 0.02 ± 0.22 m/s (10 s) Correlation coefficient: 0.96 (10 s)
Jang et al. (140)	Brachial-ankle PWV (estimated)	HealthyIndividual: 123 Average: 47	None	CV (individual pulse waves analyzed): 2.52% CV (average from several pulse waves): 0.27%
Liu et al. (121)	Heart-ear, heartfinger, heart-toe PWV (bilateral)	15 healthy	3 mo	Technical error of measurement (TEM) and relative TEM (rTEM): -heart-ear: TEM 0.005 and 0.0058, rTEM 4.7% and 5.4% -heart-finger: TEM 0.0538 and 0.0601, rTEM 1.2% and 1.2% -heart-toe: TEM 0.054 and 0.0661, rTEM 1.1% and 1.3%
Nabeel et al. (173)	Local carotid PWV	25 healthy	10 s	Correlation coefficient: 0.97 Limits of agreement: −0.01 ± 0.19 m/s
Alivon et al. (86)	Finger-toe PWV	38 unhealthy, 7 healthy	5 min	CV: 4.52% Limits of agreement: 0.02 ± 0.98 m/s
**Systolic (SBP), diastolic (DBP) and mean (MBP) blood pressure (BP)**				
Scanlon et al. (124)	Toe BP, toe-brachial index (TBI)	60 patients with diabetes	7 days	ICC and standard error of measurement (SEM): -toe BP intrarater reliability: ICC 0.78–0.79, SEM 8 mmHg -toe BP interrater reliability: ICC 0.93, SEM 4 mmHg -TBI intrarater reliability: ICC 0.51–0.72, SEM 0.08 -TBI interrater reliability: ICC 0.85, SEM 0.07
Hoyer et al. (155)	Ankle and toe SBPs	60 unhealthy	3 mo	CV of toe SBP: Vicorder device 5.63%, Falcon device 6.36% CV of ankle SBP: Vicorder device 3.43%, Falcon device 4.01%
**Single PPG pulse wave parameters**				
von Wowern et al. (139)	Finger PPG indices	112 Healthy and unhealthy	Consecutive measurements	Good repeatability (ICC ≥ 0.80): aging index (AGI), dicrotic index, dicrotic dilatation index, cardiac ejection elasticity index, *b*/*a*, *e*/*a*. Moderate repeatability (ICC: 0.50–0.79): elasticity index, *c*/*a*, *d*/*a*, *a–b* and *a–e* intervals. Poor repeatability (ICC *<* 0.50): ejection time compensated, dicrotic elasticity index, a-c and a-d intervals.
Peltokangas et al. (27)	AGI and amplitude ratios from 5 body locations	Atherosclerotic, healthy	Beat-to-beat Single session 3 days	Beat-to-beat: ICCs mostly *>*0.8. Single session: ICCs mostly *>*0.95, average intra-subject CV *<* 0.1. 3 days: ICCs for some indices *>*0.6, with few *>*0.8.
Millasseau et al. (73)	Finger stiffness index (SI)	8 healthy	1 wk	Within-subject CV: 9.6%
Millasseau et al. (91)	Finger PPG indices	8 healthy	Short-term: same day Long-term: ≥ 3 days	Within-subject CV: high short-term repeatability (*<*5%): SI, reflection index, b/a low short-term repeatability (*>*10%): *c*/*a*, *d*/*a*, *e*/*a* high long-term repeatability (*<*10%): SI, *b*/*a* low long-term repeatability (*>*10%): reflection index, *c*/*a*, *d*/*a*, *e*/*a*
Kulin et al. (85)	Finger PPG indices	Pulse wave simulator, 10 healthy	minutes	Pulse wave simulator: very low CVs (*<*1%) for all parameters in “normal” mode; high CVs (*>*10%) for AGI, *d*/*a* and *c–d* detection ratio in “abnormal” mode. Healthy subjects: low CVs (*<*5%) for left ventricular ejection time, heart rate, interbeat interval, b/a, SI; moderate CV (7.4%) for reflection index; high CVs (*>*10%) for AGI, *c–d* detection ratio, *d*/*a*.
Gunarathne et al. (108)	Finger SI	100 healthy	5 min weeks	Limits of agreement: 0.09 ± 1.32 m/s (5 min), 0.12 ± 1.86 m/s (6 wk)
**Other parameters**				
Tanaka et al. (116)	Finger MBP, finger arterial SI and elasticity index	6 healthy	day(s)	Mean CV: MBP 4.51%, SI 5.72%, elasticity index 8.20%
LopezBeltran et al. (145)	Peripheral vascular compliance index	9 healthy	Single session	CV: from 11.3% to 15.1% depending on MBP

PPG, photoplethysmogram.

Some PPG-derived parameters were found to be more stable than others, even when assessed over longer periods of time. For example, PPG-derived PWV seems to be most stable, as its short-term coefficient of variation (CV) is ∼5%, and some studies report relatively good reproducibility in the longer term (121). Toe and ankle SBPs had acceptable CVs (≤6%) even when measured 3 mo apart (155).

Parameters derived from the PPG pulse wave show similar or higher variability than PWV, with a marked difference between parameters. Parameters that are indicative of large artery stiffness (e.g., stiffness index, *b/a*, augmentation index) were more repeatable than parameters that are strongly influenced by the smaller arteries (91, 139). Most second derivative parameters (e.g., *d/a* and *e/a*) also seemed to be less repeatable. These parameters could be more sensitive to motion artifact and small changes to the input signal.

It can be difficult to assess the repeatability of PPG-derived parameters since cardiovascular properties are ever-changing even at rest (27, 170, 220, 221). To address this, the repeatability of PPG-derived parameters has been assessed using simulated signals, which allow the performance of a device to be assessed when taking repeat measurements under identical conditions. Recently, the performance of a PPG pulse wave analysis system was assessed using pulse wave simulators (85). This provides a promising approach to assess device performance directly without the influence of physiological variations.

### What is the Potential Clinical Utility of PPG-Derived Parameters of Vascular Age?

#### Key messages.

Much of the evidence for the potential clinical utility of PPG-derived parameters relates to identifying PAD, identifying diabetes, and risk prediction. Certain parameters of pulse wave shape have been found to be associated with cardiovascular risk.

The evidence is summarized in Table 8.

**Table 8.** Studies on the potential clinical utility of ppg-derived parameters of vascular age

Study	PPG Parameter	Health Status	Findings
**Identifying atherosclerosis, including peripheral arterial disease (PAD)**			
Bortolotto et al. (51)	Augmentation index (AIx), aging index (AGI)	Hypertensive, some atherosclerotic	The AGI may have some utility as a measure of atherosclerosis in older hypertensives, although carotid-femoral PWV had better performance.
Peltokangas et al. (27)	Amplitude ratios, AGI	Healthy and atherosclerotic	The AGI and some amplitude ratios measured at second toe may have utility as a measure of atherosclerosis (ROC AUC 0.79).
Allen et al. (106)	Toe pulse arrival time (PAT), shape index, rise time, amplitude	Healthy and PAD	All parameters differed between healthy and PAD. The bilateral differences in parameters (except normalized amplitude) differed between healthy and PAD.
Ro et al. (129)	Toe PPG pulse waves	Healthy and PAD	Identified PAD through manual review of toe pulse waves. This provided complementary performance to the ankle-brachial index (ABI).
Bentham et al. (23)	Variability in pulse amplitude and PAT	Healthy and PAD	Variability in amplitude reduced, and variability in PAT increased, in PAD.
Wu et al. (38)	PPG pulse wave timings	Healthy and diabetic	Results indicated that PPG pulse wave timings could be used to discriminate between healthy and diabetic subjects.
**Risk prediction**			
Kuznetsova et al. (82)	Pulse amplitude after occlusion	Population cohort	Change in pulse amplitude after occlusion correlated weakly with cardiovascular risk factors.
Inoue et al. (169)	*d/a*	Population cohort	*d*/*a* found to be an independent predictor of cardiovascular mortality.
Zekavat et al. (49)	Stiffness index (SI)	Population cohort	SI found to be a genetically causal risk factor for blood pressure but not coronary artery disease.
Gunarathne et al. (108)	SI	Healthy, hypertensive, diabetic, hyperlipidemic	SI was associated with cardiovascular risk (HeartScore) and able to discriminate between risk categories.
**Identifying and stratifying subjects with diabetes**			
Wei et al. (25)	SI, instantaneous energy of maximal energy (*f*_Emax_)	Healthy, diabetic	SI and *f*_Emax_ were higher in diabetic subjects than age-matched healthy subjects. *f*_Emax_ was associated with glycated hemoglobin levels (indicative of how well diabetes is controlled) and fasting blood sugar levels.
Wu et al. (159)	Pulse amplitudes, pulse wave velocity (PWV) (bilateral)	Healthy, diabetic	Bilateral differences in pulse amplitudes and PWV were sensitive to elevated glycated hemoglobin levels, and were correlated with cardiovascular risk factors.
Usman et al. (132)	Area under the pulse wave	Diabetic	The area under the pulse wave was lower in patients with higher glycated hemoglobin levels (and higher risk of complications).
Pilt et al. (90)	AGI	Healthy and diabetic	AGI higher in diabetic subjects than age-matched healthy subjects.
Pilt et al. (135)	AIx	Healthy and diabetic	AIx higher in diabetic subjects than age-matched healthy subjects.
Pilt et al. (133)	Slope of the rising front (ms)	Healthy and diabetic	The slope of the rising front can be used to discriminate between healthy and diabetic subjects.
**Miscellaneous**			
Wang et al. (119)	Finger-toe PWV, compliance index (CI)	Healthy and chronic kidney disease	CI and PWV differed between healthy subjects and chronic kidney disease patients, and changed with disease progression. A decrease in CI was associated with an increase in the number of cardiovascular risk factors. CI was independently associated with estimated glomerular filtration rate.
Sangle et al. (130)	SI	Healthy and patients with livedo	No difference in SI between groups despite more abnormal carotid-femoral PWV in patients with livedo.
von Wowern et al. (36)	AGI, *b/a*, *d/a*	Pregnant women	Parameters changed during pregnancy, but variance was greater than the influence of gestational age.
Bereksi-Reguig et al. (166)	AIx, *b/a*	Healthy and pathologic	Both PPG-derived parameters differed between normal and pathological subjects.
Sharkey et al. (24)	Toe-finger, toe-ear, finger-ear pulse transit time (PTT)	Children: healthy and heart transplant	PTT was increased in children who have successfully undergone cardiac transplantation.
Dillon and Hertzman (101)	Crest time (CT)	Healthy, hypertensive, and arteriosclerotic	Crest time was increased in hypertensive and arteriosclerotic patients. Changes were greater, and visible at an earlier stage of disease, in finger compared to radial PPG signals.
Tanaka et al. (116)	Finger arterial SI	Healthy, arteriosclerotic	The SI appeared to be higher in arteriosclerotic subjects.
Kiselev and Karavaev (81)	Indices of frequency spectral power	Healthy, hypertensive, coronary artery disease	High frequency power (HF%, 0.15–0.40 Hz) increased in disease; low frequency power (LF%, 0.04–0.15 Hz) and LF/HF decreased.

PPG, photoplethysmogram.

Several studies have investigated the potential clinical utility of PPG-based approaches for identifying PAD (23, 62, 106, 129, 155) using a range of parameters derived from toe PPG: pulse wave features, variability in PAT and pulse wave features, and systolic BP. Current evidence indicates that these approaches may be complementary to the ABI (such as providing increased sensitivity and decreased specificity) (129). Future research should establish which PPG-based approach provides the best performance, and to assess its clinical utility in comparison to the ABI, which is routinely used in clinical practice.

PPG-derived parameters may have utility for cardiovascular risk prediction. *d/a* has been found to be predictive of cardiovascular mortality independently of age, BP, and other atherosclerosis-related factors (169). The stiffness index has been found to be a causal risk factor for elevated BP (49), which in turn confers increased cardiovascular risk (179). The stiffness index has also been found to be associated with cardiovascular risk (108). Future research should compare the predictive performance of a wide range of PPG-derived parameters to establish which provides best performance independent of existing risk factors.

PPG-derived parameters may have utility for identifying diabetes, and for assessing how well it is controlled. Several PPG-derived parameters have been found to differ between subjects with diabetes and healthy subjects, potentially providing opportunity to identify early signs of diabetes, which can remain undiagnosed for several years (132). In addition, PPG-derived parameters have been found to be associated with glycated hemoglobin levels in patients with diabetes, providing opportunity to identify patients whose diabetes is less well controlled, and who are at greater risk of complications. It has been proposed that such approaches could be incorporated into smartphones for widespread use (222).

PPG-derived parameters may also have utility for identifying increased arterial stiffness or BP in pregnancy which can precede preeclampsia (36). It is important to identify preeclampsia early as it can result in maternal and fetal morbidity and mortality (223). PPG-equipped wearables such as smartwatches and fitness trackers present a potential approach for doing this. Current evidence on PPG-derived parameters is limited to complication-free pregnancies (36), and further research should investigate whether parameters change consistently before preeclampsia.

### What Resources Are Available to Researchers?

#### Key messages.

Several openly available data sets are available that contain PPG signals. However, most are only suitable for assessing techniques which use a single PPG signal to estimate BP, or for investigating associations between PPG-derived parameters and chronological age.

This review identified six openly available data sets that have been used to assess PPG-derived parameters of vascular age, as summarized in Table A4. All the data sets contain a PPG signal and reference BP values, so are suitable for assessing techniques to estimate BP from a single PPG signal. Most data sets include subjects’ chronological ages. The MIMIC and University of Queensland datasets also contain ECG signals, so may be suitable for assessing techniques that use both PPG and ECG signals (although the time delay between ECG and PPG signals in the MIMIC database is not necessarily constant, even within a particular subject) (231). The MIMIC data set also contains reference ABI measurements. The Pulse Wave Database contains simulated PPG pulse waves (4), so does not permit in vivo assessments, but can be used to assess how simulated pulse waves vary under a range of cardiovascular conditions (including changes in arterial stiffness) for subjects aged from 25 to 75. Consequently, it may be helpful for initial assessment of techniques, and also to aid the design of in vivo studies (4). Additional openly available data sets would be highly valuable for future research, ideally containing simultaneous ECG signals and PPG signals at different sites, and reference indicators of vascular age (such as PWV, BP, and PAD diagnoses).

There is relatively little code available to estimate parameters of vascular age from the PPG. Only one publication was identified for which the related code is publicly available (41). The “PulseAnalyse” tool (4, 232) may also be of use to researchers, as it *1*) identifies individual pulse waves in PPG signals, *2*) identifies fiducial points on pulse waves, and *3*) derives feature measurements suitable for assessing vascular age. In addition, “TTalgorithm” is a helpful script for extracting PTT measurements from simultaneous pulse wave signals (233). In the future, the field would benefit greatly from researchers making their analysis code publicly available, aiding reproducibility, and allowing others to build on their work. The Papers with code website (https://portal.paperswithcode.com/) may be helpful for this, which facilitates sharing of code and data, and has a leaderboard of techniques used to estimate BP from the PPG (see https://paperswithcode.com/task/blood-pressure-estimation).

In the future, data sets containing annotations of fiducial points on PPG pulse waves could be used to assess pulse wave analysis algorithms. We are not aware of any data sets containing manual annotations of fiducial points, although the Pulse Wave Database does contain machine-generated annotations (4).

### Future Research Directions

#### Key messages.

There is much further work to be done to realize the full potential of PPG-based devices for assessing vascular age. Several directions have been identified in the areas of *1*) science and technology of measurements, *2*) clinical considerations and wider validation, and *3*) sensors and computing science.

This review has demonstrated the great potential of using the PPG to assess vascular age. A resurgence of interest in the PPG has been driven by: the demand for low-cost, simple, and portable technology suitable for primary care and community-based clinical settings; the availability of low-cost and miniature semiconductor components; and advances in pulse wave analysis techniques. Interest in the PPG has continued to grow with the development of: state-of-the-art wearable sensors; digital health and smartphone platforms; ML and artificial intelligence (AI) based analysis techniques; and cloud-based analytics. The Covid-19 pandemic appears to have accelerated developments in healthcare technologies, including PPG devices and their applications (234, 235). Despite such innovation, several research questions remain unanswered, as now outlined. Future research into using the PPG to assess vascular age will benefit from interdisciplinary collaboration to address these questions, often requiring consensus agreement.

#### Science and technology of measurements.

- *Physiological origins:* To better understand the origins of the PPG signal, since its physiological origins are still not well understood (236). The origins of both the “AC” pulse wave and the lower frequency “DC” components should be further investigated.
- *Measurement and analysis technology:* To further understand the influence of technical factors such as operating wavelength(s), probe-tissue interface pressure, sampling frequency, the mode of PPG used (reflection or transmission), and the acquisition of PPG signals by contact or noncontact (“imaging”) methods.
- *Reproducibility:* To use an agreed measurement procedure that has been demonstrated to improve reproducibility in studies, including specifications such as: body position, anatomical measurement site, duration of measurement, PPG sensor configuration, and signal processing techniques. To openly share repeatability and reproducibility data in publications as standard. To gather a minimum data set that includes participant demographics and key measurement parameters used for the PPG recording.
- *Normal ranges:* To establish normal ranges for PPG-derived parameters at different body sites and across different populations, including stratification by sex, skin pigmentation, and chronological age.
- *Signal quality:* To standardize signal quality indices, and optimize the design of algorithms for PPG noise and artifact rejection. Furthermore, the potential benefits of acquiring other signals simultaneously to enhance or monitor PPG signal quality should be further investigated, such as accelerometer or gyroscope signals for noise detection and cancellation (92).
- *Understanding variability:* To understand the variability in PPG-derived parameters. To understand normal physiological variability (such as short-term variability during a measurement and diurnally) so that average “representative” measures can be extracted for analysis. To understand the influence of cardiac arrhythmias such as atrial fibrillation, and their impact on vascular aging assessments. To understand the utility of PPG-derived parameters acquired during sleep, which are less likely to be contaminated with motion artifact (237), but may not provide the wealth of information afforded by measurements taken during activities of daily living (238).

#### Clinical considerations and wider validation.

- *Systematic assessment of existing parameters:* To systematically assess existing PPG-derived parameters of vascular aging (e.g., the stiffness index and those derived from the second derivative) to understand which parameters (or combinations of parameters) are most suitable for assessing vascular age.
- *Physiological determinants:* To understand further the influence of confounding factors on PPG-derived parameters, such as the influences of heart rate and BP.
- *Microcirculation:* To understand further the impact of the microcirculation on PPG-derived parameters, and determine how we might use this insight to improve assessments. Although several methods are available for assessing arterial stiffness, the PPG signal has a particular but seldom explored advantage, that it can provide composite macrovascular and microvascular information. To date, most techniques for assessing vascular age from the PPG focus on the larger blood vessels. The microcirculation may also provide useful information, and can be assessed in a multisite PPG configuration (using bilateral and ipsilateral body site comparisons) (205). Strict protocols should be employed for microvascular measurements, considering: a rest period before measurements; room temperature; and careful handling of tissue sites so as not to invoke perfusion changes. Also, the potential utility of PPG-derived measures of endothelial and autonomic function (239) should be investigated, and their associations with arterial stiffness and atherosclerosis measures.
- *Validating parameters:* To develop and clinically validate PPG-derived parameters of vascular age against a recognized gold standard for “vascular age,” rather than chronological age. The ultimate goal should be to develop and validate PPG-based biomarkers of vascular age (185, 240).
- *Assessing reliability:* To assess the reliability of PPG-derived parameters of vascular age, i.e., the relative magnitude of measurement errors in comparison to true differences between subjects (219).
- *Unobtrusive monitoring in daily life:* Approaches which use a single PPG signal could be incorporated into wearables such as smartwatches, providing opportunity to assess vascular age in large numbers of subjects in daily life. Although such an approach may not provide a gold-standard assessment of vascular age, it could be complementary to the current gold standard of carotid-femoral pulse wave velocity: the sensors would be less expensive; measurements would not require a trained operator; and by taking measurements during sleep, measurements could be standardized (same time of day, and supine position), and the subject would not need to set aside time to rest before measurement. To achieve this, technologies should be robust and resilient to movement artifact and noise, incorporated into consumer devices, and ideally have applications in health and wellbeing. Such measurements could be incorporated into wrist-worn devices, finger probes, and weighing scales for longitudinal assessments easily made in the home (65).

#### Sensors and computing science.

- *Sensors:* To develop novel PPG sensors for the specific goal of assessing vascular age. This should consider also the attachment of sensor to tissue for repeatable measurements, for example to stabilize the contact by applying an external pressure (76, 78). PPG sensors should be designed to mitigate against probe-tissue signal artifacts and to help obtain high-quality signals (241). Ideally, PPG sensors should be standardized to ensure measurements are replicable.
- *Artificial intelligence:* To exploit the power of AI (including explainable AI) and potentially quantum healthcare technologies for PPG processing and to help model and understand big data sets stemming from worldwide sampling.

## CONCLUSIONS

The PPG signal is emerging as a potential tool for assessing vascular age, with potential applications in clinical and consumer devices. The shape and timing of the PPG pulse wave are both influenced by normal vascular aging, changes in arterial stiffness and blood pressure, and atherosclerosis. Consequently, a plethora of approaches have been proposed to assess vascular age from the PPG. These approaches fall into three categories: *1*) those which use a single PPG signal (based on pulse wave analysis), *2*) those which use multiple PPG signals (e.g., PTT measurement), and *3*) those which use PPG and other signals (e.g., PAT measurement). There is evidence in the literature on the level of agreement between PPG-derived parameters and reference indicators of vascular age, and on the repeatability and reproducibility of selected parameters. Furthermore, the clinical utility of PPG-derived parameters has been explored in the fields of PAD, diabetes, and cardiovascular risk prediction. However, there is much further work to be done to realize the full potential of PPG-based devices for assessing vascular age.

Key directions for future work include:

- Gaining a better understanding of the physiological origins of the PPG signal, and how it is influenced by the stiffness of large and small arteries.
- Standardizing measurement techniques to ensure that PPG-derived parameters are measured robustly, both for clinical decision making and in the rapidly growing consumer market.
- Validating PPG-based techniques for assessing vascular age, and assessing their potential clinical utility.

**Figure 6.**
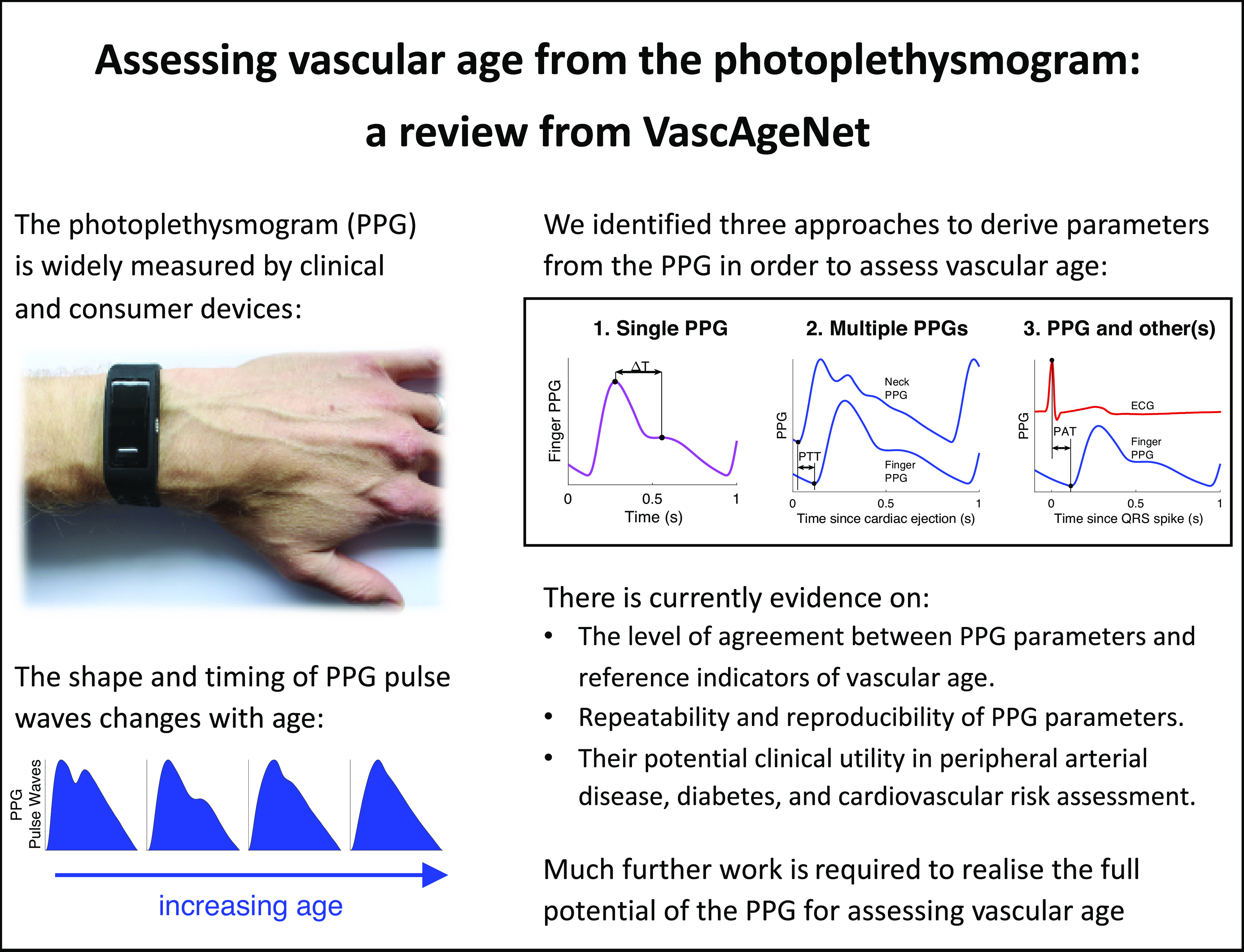
A graphical summary of the key conclusions. Wristband adapted from P. H. Charlton, “Max Health Band” (CC BY 4.0). Pulse waves adapted from: P. H. Charlton, “Classes of photoplethysmogram (PPG) pulse wave shape” (CC BY 4.0).

Figure 6 provides a graphical summary of these conclusions.

## GRANTS

This article is based upon work from COST ACTION “Network for Research in Vascular Ageing” CA18216 supported by COST (European Cooperation in Science and Technology): www.cost.eu. The work was supported in part by British Heart Foundation Grants PG/15/104/31913 and FS/20/20/34626 (to P. H. Charlton); in part by the European Regional Development Fund Project No. 01.2.2-LMT-K-718-01-0030 (to V. Marozas) under grant agreement with the Research Council of Lithuania; in part by the Estonian Ministry of Education and Research under personal post-doctoral research funding PUTJD815 (to K. Pilt); and in part by the Serbian Ministry of Education, Science and Technological Development Grants 32040 and 41022 (to D. Žikić).

## DISCLOSURES

S. Zanelli collaborates with Axelife, a company that designs and develops PPG-based medical devices. D. Kulin is shareholder and employee in E-Med4All Europe Ltd., a Hungarian med-tech startup developing various PPG-based telemedicine solutions. M. Hallab is CEO of Axelife and has authored patents used by Axelife. E. Bianchini is co-founder of QUIPU s.r.l., Pisa, Italy, a spin-off company of the Italian National Research Council and the University of Pisa developing medical software for ultrasound image processing. V. Dittrich is CEO and shareholder of Redwave Medical GmbH, a company developing medical algorithms for pulse wave analysis. None of the other authors has any conflicts of interest, financial or otherwise, to disclose.

## AUTHOR CONTRIBUTIONS

P.H.C., B.P., M.B., M.H., V.D., B.H., D.V., D.Ž., and V.M. conceived and designed research; P.H.C., B.P., K.P., M.B., S.Z., D.K., D.Ž., and V.M., performed experiments; P.H.C., B.P., K.P., M.B., S.Z., D.K., D.Ž., and V.M. analyzed data; P.H.C., B.P., K.P., M.B., S.Z., D.K., J.A., D.V., D.Ž., and V.M. interpreted results of experiments; P.H.C., K.P., M.B. D.Ž., and V.M. prepared figures; P.H.C., B.P., K.P., M.B., S.Z., D.K., J.A., V.D., D.V., D. Ž., and V.M., drafted manuscript; P.H.C., B.P., K.P., M.B., S.Z., D.K., J.A., E.B., C.C.M., D.T.-P., B.H., D.V., D.Ž., and V.M. edited and revised manuscript; P.H.C., B.P., K.P., M.B., S.Z., D.K., J.A., M.H., E.B., C.C.M., D.T.-P., V.D., B.H., D.V., D.Ž., and V.M., approved final version of manuscript.

## APPENDIX

### Search Methodology

#### Search commands.

The search commands used with each search engine were as follows:

##### ACM digital library.

The ACM Digital Library Advanced Search (https://dl.acm.org/search/advanced) was used to search the “ACM Guide to Computing Literature” with the following query:

*Title:(photoplethysmogra* OR ppg OR “pulse contour” OR “volume pulse” OR “volume wave”) AND Title:(age OR ageing OR aging OR BP OR “decomposition analysis” OR elasticity OR hypertension OR “intensity analysis” OR PAT OR PDA OR peripheral OR PWV OR pressure OR PTT OR “pulse arrival time” OR “pulse transit time” OR “pulse wave velocity” OR stiffness OR “time difference”)*

##### IEEE xplore.

The IEEE Xplore “Command Search (https://ieeexplore.ieee.org/search/advanced/command)” was used to search with the following query:

*(((((“Document Title”:photoplethysmogra*) OR “Document Title”:ppg) OR “Document Title”:”pulse contour”) OR “Document Title”:”volume pulse”[Title]) OR “Document Title”:”volume wave”[Title]) AND (((((((((((((((((((“Document Title”:age) OR “Document Title”:ageing) OR “Document Title”:aging) OR “Document Title”:BP) OR “Document Title”:”decomposition analysis”) OR “Document Title”:elasticity) OR “Document Title”:hypertension) OR “Document Title”:”intensity analysis”) OR “Document Title”:PAT) OR “Document Title”:PDA) OR “Document Title”:peripheral) OR “Document Title”:PWV) OR “Document Title”:pressure) OR “Document Title”:PTT) OR “Document Title”:”pulse arrival time”) OR “Document Title”:”pulse transit time”) OR “Document Title”:”pulse wave velocity”) OR “Document Title”:stiffness) OR “Document Title”:”time difference”)*

##### PubMed.

The “PubMed Advanced Search Builder (https://pubmed.ncbi.nlm.nih.gov/advanced/)” was used to search with the following query:

*(((((photoplethysmogra*[Title]) OR ppg[Title]) OR “pulse contour”[Title]) OR “volume pulse”[Title]) OR “volume wave”[Title]) AND (((((((((((((((((((age[Title]) OR ageing[Title]) OR aging[Title]) OR BP[Title]) OR “decomposition analysis”[Title]) OR elasticity[Title]) OR hypertension[Title]) OR “intensity analysis”[Title]) OR PAT[Title]) OR PDA[Title]) OR peripheral[Title]) OR PWV[Title]) OR pressure[Title]) OR PTT[Title]) OR “pulse arrival time”[Title]) OR “pulse transit time”[Title]) OR “pulse wave velocity”[Title]) OR stiffness[Title]) OR “time difference”[Title])*

##### Scopus.

The Scopus “Advanced Search (https://www.scopus.com/search/form.uri?display=advanced)” was used to search with the following query:

*TITLE(photoplethysmogra* OR ppg OR “pulse contour” OR “volume pulse” OR “volume wave”) AND TITLE(age OR ageing OR aging OR BP OR “decomposition analysis” OR elasticity OR hypertension OR “intensity analysis” OR PAT OR PDA OR peripheral OR PWV OR pressure OR PTT OR “pulse arrival time” OR “pulse transit time” OR “pulse wave velocity” OR stiffness OR “time difference”) AND NOT INDEX(medline)*

##### Web of science.

The Web of Science “Advanced Search” was used to search the “Web of Science Core Collection” with the following query:

*TI=(photoplethysmogra* OR ppg OR “pulse contour” OR “volume pulse” OR “volume wave”) AND TI=(age OR ageing OR aging OR BP OR “decomposition analysis” OR elasticity OR hypertension OR “intensity analysis” OR PAT OR PDA OR peripheral OR PWV OR pressure OR PTT OR “pulse arrival time” OR “pulse transit time” OR “pulse wave velocity” OR stiffness OR “time difference”)*

#### Collating search results.

Results from each search engine were downloaded as text files. Files were obtained in Bibtex format for all search engines apart from PubMed, for which the “*Abstract (text)*” format was used. The results were then collated using the *ppg_vascage_review_collate_search_data.m* MATLAB script, available in Supplemental material at https://doi.org/10.5281/zenodo.5039640.

#### Removing duplicates.

Duplicate publications were identified as any publications with the same DOI; any publications with the same title and other consistent details (such as authors and year of article). A few additional duplicates were identified using the Rayyan web application (16).

#### Additional publications.

Four additional publications were added to the manual search during the course of the review (82, 83, 85, 86).

### Distribution of Articles According to Publication Year

The distribution of articles according to publication year is presented in Fig. A1, and discussed in *Source of Evidence*.

### Approaches Used to Assess Indicators of Vascular Age

The approaches used to assess the different indicators of vascular age are summarized in Table A1, and discussed in *What Indicators of Vascular Age Have Been Assessed?* and in *How Have Indicators of Vascular Age Been Derived?*

### Methods Used to Assess the Performance of PPG-Derived Parameters of Vascular Age

The characteristics of the subjects in the included studies of PPG-derived parameters of vascular age are summarized in Table A2, and discussed in *How Has the Performance of PPG-Derived Parameters of Vascular Age Been Assessed?*

Key aspects of the experimental methodologies used to assess PPG-derived parameters of vascular age are summaries in Table A3, and discussed in *How Has the Performance of PPG-Derived Parameters of Vascular Age Been Assessed?*

### Methods Used to Assess the Performance of PPG-Derived Parameters of Vascular Age

The openly available data sets that have been used to assess PPG-derived parameters of vascular age are summarized in Table A4, and discussed in *What Resources are Available to Researchers?*

**Table A1.** The number of articles which used each approach to assess vascular age, and which assessed each indicator of vascular age

	Blood Pressure	Stiffness	Atherosclerosis	Chronological Age	Utility	All
Single PPG	50	15	2	17	11	135
Multiple PPG	11	3	3	1	4	33
PPG and Other(s)	41	9	2	2	7	59
All	95	26	4	20	21	

*NB*: Some articles used more than one approach or one indicator. PPG, photoplethysmogram.

**Table A2.** Characteristics of participants in studies of PPG-derived parameters of vascular age

Category	No. Articles (%)
Number of subjects	
≤9	8 (4.9)
10–49	65 (40.1)
50–99	32 (19.8)
100–499	43 (26.5)
500–999	6 (3.7)
**≥**1,000	7 (4.3)
Unknown	1 (0.6)
Age(s) of subjects, yr	
≤17: Pediatric	6 (3.7)
18–39: Young adult	104 (64.2)
40–69: Middle-aged adult	107 (66.0)
≥70: Elderly adult	73 (45.1)
Unknown	30 (18.5)
Proportion of subjects who were female, %	
0–20: Mostly male	22 (13.6)
21–40: Primarily male	23 (14.2)
41–60: Well balanced	46 (28.4)
61–80: Primarily female	10 (6.2)
80–100: Mostly female	6 (3.7)
Unknown	55 (33.0)
Most common health statuses	
Healthy	118 (72.8)
Unhealthy (nonspecific)	24 (14.8)
Critically ill	17 (10.5)
Hypertensive	17 (10.5)
Diabetic	15 (9.3)
Peripheral arterial disease	8 (4.9)
Population cohort	4 (2.5)
Under anesthesia	4 (2.5)

**Table A3.** Methods used to assess the performance of PPG-derived parameters of vascular age

Category	No. Articles (%)
Reference indicator of vascular age	
Blood pressure: noninvasive	60 (37.0)
Blood pressure: invasive	23 (14.2)
Chronological age (a surrogate)	20 (12.3)
Ankle-brachial index	12 (7.4)
Pulse wave velocity: carotid-femoral	11 (6.8)
Pulse wave velocity: other paths	5 (3.1)
Pulse arrival time	3 (1.9)
Pulse transit time	1 (0.6)
Other stiffness indices, e.g., AIx	6 (3.7)
None	21 (13.0)
Common statistical measures	
Correlation coefficient	75 (46.3)
Bias + limits of agreement	54 (33.3)
Mean absolute (percentage) error	23 (14.2)
Root-mean-square error (RMSE)	14 (8.6)
Classification statistics, e.g., sens, spec	20 (12.3)
Number of datasets used	
1	141 (87.0)
2	15 (9.3)
≥ 3	6 (3.7)

AIx, augmentation index.

**Table A4.** Datasets of PPG signals used to assess PPG-derived parameters of vascular age [Modified from (226)]

Dataset	Reference	Signals	Reference Parameters	No. Subjects	Description
UK Biobank	(224)	PPG	Blood pressure (BP), chronological age	205,337	Single finger PPG waves from middle-aged subjects. The stiffness index, calculated by the PPG device, is also available.
MIMIC	(225)	PPG, BP, electrocardiogram (ECG), others	BP, ankle-brachial index, chronological age	10,000	Recordings from critically ill adults and neonates, lasting from minutes to days. Typically, at finger.
Cuffless BP Estimation	(226, 227)	PPG, ECG	BP	942	Recordings from critically ill patients, each lasting ≥10 min. Extracted from the MIMIC-II Database.
PPG-BP Database	(228)	PPG	BP, chronological age	219	Three finger recordings from adults aged 20–89 with and without cardiovascular disease, three waves per recording.
University of Queensland Vital Signs Dataset	(229)	PPG, ECG, BP	BP	32	Recordings from patients during anesthesia, ranging from minutes to hours in duration.
Pulse Wave Database	(4, 230)	PPG, BP	BP, pulse wave velocity, chronological age, others	4,374(simulated)	Single simulated PPG pulse waves representative of healthy adults aged 25–75.

BP, blood pressure; PPG, photoplethysmogram
