# Supplementary material for: Assessing hemodynamics from the photoplethysmogram to gain insights into vascular age: a review from VascAgeNet
Source: Am J Physiol Heart Circ Physiol. Author manuscript; Available in PMC 2022 Apr 1. (PMC8917928; doi:10.1152/ajpheart.00392.2021)
Supplement: Appendix [file EMS143851-supplement-Appendix.pdf]

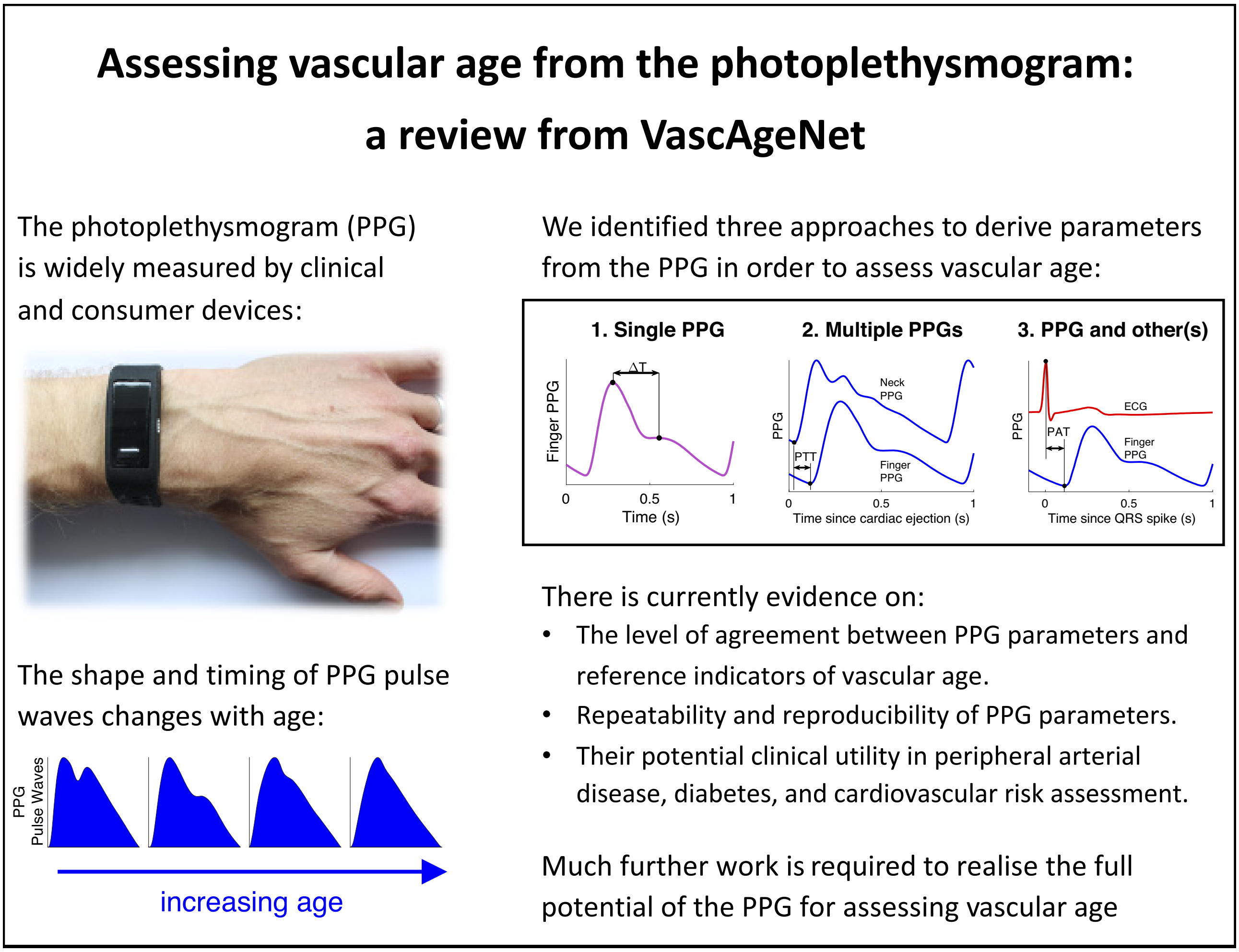

Figure 6. A graphical summary of the key conclusions. Wristband adapted from P. H. Charlton, "Max Health Band" (CC BY 4.0). Pulse waves adapted from: P. H. Charlton, "Classes of photoplethysmogram (PPG) pulse wave shape" (CC BY 4.0).

(based on pulse wave analysis), *2*) those which use multiple PPG signals (e.g., PTT measurement), and *3*) those which use PPG and other signals (e.g., PAT measurement). There is evidence in the literature on the level of agreement between PPG-derived parameters and reference indicators of vascular age, and on the repeatability and reproducibility of selected parameters. Furthermore, the clinical utility of PPG-derived parameters has been explored in the fields of PAD, diabetes, and cardiovascular risk prediction. However, there is much further work to be done to realize the full potential of PPG-based devices for assessing vascular age.

Key directions for future work include:

- Gaining a better understanding of the physiological origins of the PPG signal, and how it is influenced by the stiffness of large and small arteries.
- Standardizing measurement techniques to ensure that PPG-derived parameters are measured robustly, both for clinical decision making and in the rapidly growing consumer market.
- Validating PPG-based techniques for assessing vascular age, and assessing their potential clinical utility.

Figure 6 provides a graphical summary of these conclusions.

APPENDIX

Search Methodology

Search commands.

The search commands used with each search engine were as follows:

ACM digital library. The ACM Digital Library Advanced Search (https://dl.acm.org/search/advanced) was used to search the "ACM Guide to Computing Literature" with the following query:

Title:(photoplethysmogra OR ppg OR "pulse contour" OR "volume pulse" OR "volume wave") AND Title:(age OR ageing OR aging OR BP OR "decomposition analysis" OR elasticity OR hypertension OR "intensity analysis" OR PAT OR PDA OR peripheral OR PWV OR pressure OR PTT OR "pulse arrival time" OR "pulse transit time" OR "pulse wave velocity" OR stiffness OR "time difference")*

IEEE xplore. The IEEE Xplore "Command Search (https://ieeexplore.ieee.org/search/advanced/command)" was used to search with the following query:

((((("Document Title":photoplethysmogra) OR "Document Title":ppg) OR "Document Title":"pulse contour") OR "Document Title":"volume pulse"[Title]) OR "Document*

Downloaded from journals.physiology.org/journal/ajpheart (080.189.253.250) on March 11, 2022.

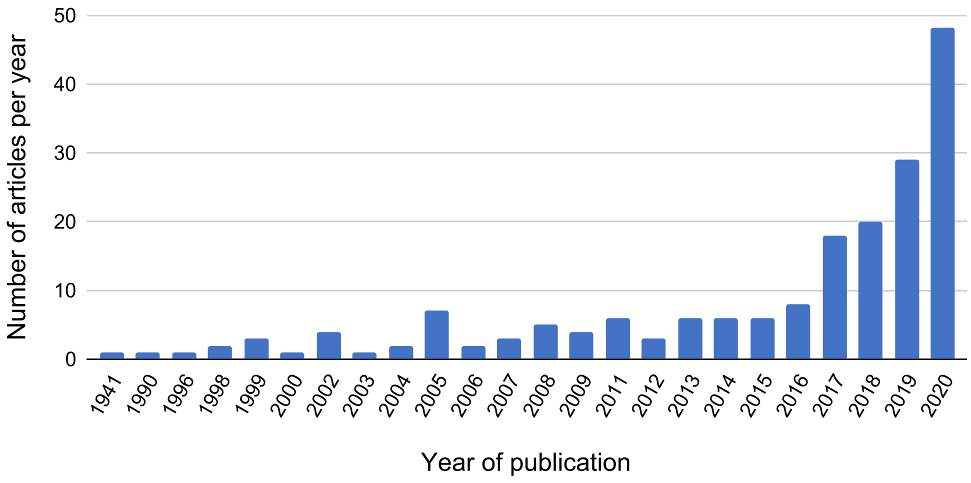

Figure A1. The number of included articles published per year.

Title":"volume wave"[Title]) AND ((((((((((((((((("Document Title":age) OR "Document Title":ageing) OR "Document Title":aging) OR "Document Title":BP) OR "Document Title":"decomposition analysis") OR "Document Title":elasticity) OR "Document Title":hypertension) OR "Document Title":"intensity analysis") OR "Document Title":PAT) OR "Document Title":PDA) OR "Document Title":peripheral) OR "Document Title":PWV) OR "Document Title":pressure) OR "Document Title":PTT) OR "Document Title":"pulse arrival time") OR "Document Title":"pulse transit time") OR "Document Title":"pulse wave velocity") OR "Document Title":stiffness) OR "Document Title":"time difference")

PubMed. The "PubMed Advanced Search Builder (https://pubmed.ncbi.nlm.nih.gov/advanced/)" was used to search with the following query:

(((((photoplethysmogra[Title]) OR ppg[Title]) OR "pulse contour"[Title]) OR "volume pulse"[Title]) OR "volume wave"[Title]) AND ((((((((((((((((((age[Title]) OR ageing[Title]) OR aging[Title]) OR BP[Title]) OR "decomposition analysis"[Title]) OR elasticity[Title]) OR hypertension[Title]) OR "intensity analysis"[Title]) OR PAT[Title]) OR PDA[Title]) OR peripheral [Title]) OR PWV[Title]) OR pressure[Title]) OR PTT[Title]) OR "pulse arrival time"[Title]) OR "pulse transit time"[Title]) OR "pulse wave velocity"[Title]) OR stiffness[Title]) OR "time difference"[Title])*

Scopus. The Scopus "Advanced Search (https://www.scopus.com/search/form.uri?display=advanced)" was used to search with the following query:

TITLE(photoplethysmogra OR ppg OR "pulse contour" OR "volume pulse" OR "volume wave") AND TITLE(age OR ageing OR aging OR BP OR "decomposition analysis" OR elasticity OR hypertension OR "intensity analysis" OR PAT OR PDA OR peripheral OR PWV OR pressure OR PTT OR "pulse arrival time" OR "pulse transit time" OR "pulse wave velocity" OR stiffness OR "time difference") AND NOT INDEX (medline)*

Web of science. The Web of Science "Advanced Search" was used to search the "Web of Science Core Collection" with the following query:

TI=(photoplethysmogra OR ppg OR "pulse contour" OR "volume pulse" OR "volume wave") AND TI=(age OR ageing OR aging OR BP OR "decomposition analysis" OR elasticity OR hypertension OR "intensity analysis" OR PAT OR PDA OR peripheral OR PWV OR pressure OR PTT OR "pulse arrival time" OR "pulse transit time" OR "pulse wave velocity" OR stiffness OR "time difference")*

Collating search results.

Results from each search engine were downloaded as text files. Files were obtained in Bibtex format for all search engines apart from PubMed, for which the "*Abstract (text)*" format was used. The results were then collated using the *ppg_vascage_review_collate_search_data.m* MATLAB script, available in Supplemental material at https://doi.org/10.5281/zenodo.5039640.

Removing duplicates.

Duplicate publications were identified as any publications with the same DOI; any publications with the same title and other consistent details (such as authors and year of article). A few additional duplicates were identified using the Rayyan web application (16).

Table A1. *The number of articles which used each approach to assess vascular age, and which assessed each indicator of vascular age*

	Blood Pressure	Stiffness	Atherosclerosis	Chronological Age	Utility	All
Single PPG	50	15	2	17	11	135
Multiple PPG	11	3	3	1	4	33
PPG and Other(s)	41	9	2	2	7	59
All	95	26	4	20	21	

NB: Some articles used more than one approach or one indicator. PPG, photoplethysmogram.

Downloaded from journals.physiology.org/journal/ajpheart (080.189.253.250) on March 11, 2022.

Table A2. *Characteristics of participants in studies of PPG-derived parameters of vascular age*

Category	No. Articles (%)
Number of subjects	
≤9	8 (4.9)
10–49	65 (40.1)
50–99	32 (19.8)
100–499	43 (26.5)
500–999	6 (3.7)
≥1,000	7 (4.3)
Unknown	1 (0.6)
Age(s) of subjects, yr	
≤17: Pediatric	6 (3.7)
18–39: Young adult	104 (64.2)
40–69: Middle-aged adult	107 (66.0)
≥70: Elderly adult	73 (45.1)
Unknown	30 (18.5)
Proportion of subjects who were female, %	
0–20: Mostly male	22 (13.6)
21–40: Primarily male	23 (14.2)
41–60: Well balanced	46 (28.4)
61–80: Primarily female	10 (6.2)
80–100: Mostly female	6 (3.7)
Unknown	55 (33.0)
Most common health statuses	
Healthy	118 (72.8)
Unhealthy (nonspecific)	24 (14.8)
Critically ill	17 (10.5)
Hypertensive	17 (10.5)
Diabetic	15 (9.3)
Peripheral arterial disease	8 (4.9)
Population cohort	4 (2.5)
Under anesthesia	4 (2.5)

Additional publications.

Four additional publications were added to the manual search during the course of the review (82, 83, 85, 86).

Distribution of Articles According to Publication Year

The distribution of articles according to publication year is presented in Fig. A1, and discussed in *Source of Evidence*.

Approaches Used to Assess Indicators of Vascular Age

The approaches used to assess the different indicators of vascular age are summarized in Table A1, and discussed in *What Indicators of Vascular Age Have Been Assessed?* and in *How Have Indicators of Vascular Age Been Derived?*

Methods Used to Assess the Performance of PPG-Derived Parameters of Vascular Age

The characteristics of the subjects in the included studies of PPG-derived parameters of vascular age are summarized in Table A2, and discussed in *How Has the Performance of PPG-Derived Parameters of Vascular Age Been Assessed?*

Key aspects of the experimental methodologies used to assess PPG-derived parameters of vascular age are summaries in Table A3, and discussed in *How Has the Performance of PPG-Derived Parameters of Vascular Age Been Assessed?*

Table A3. *Methods used to assess the performance of PPG-derived parameters of vascular age*

Category	No. Articles (%)
Reference indicator of vascular age	
Blood pressure: noninvasive	60 (37.0)
Blood pressure: invasive	23 (14.2)
Chronological age (a surrogate)	20 (12.3)
Ankle-brachial index	12 (7.4)
Pulse wave velocity: carotid-femoral	11 (6.8)
Pulse wave velocity: other paths	5 (3.1)
Pulse arrival time	3 (1.9)
Pulse transit time	1 (0.6)
Other stiffness indices, e.g., AIx	6 (3.7)
None	21 (13.0)
Common statistical measures	
Correlation coefficient	75 (46.3)
Bias + limits of agreement	54 (33.3)
Mean absolute (percentage) error	23 (14.2)
Root-mean-square error (RMSE)	14 (8.6)
Classification statistics, e.g., sens, spec	20 (12.3)
Number of datasets used	
1	141 (87.0)
2	15 (9.3)
≥ 3	6 (3.7)

AIx, augmentation index.

Methods Used to Assess the Performance of PPG-Derived Parameters of Vascular Age

The openly available data sets that have been used to assess PPG-derived parameters of vascular age are summarized in Table A4, and discussed in *What Resources are Available to Researchers?*

GRANTS

This article is based upon work from COST ACTION "Network for Research in Vascular Ageing" CA18216 supported by COST (European Cooperation in Science and Technology): www.cost.eu. The work was supported in part by British Heart Foundation Grants PG/15/104/31913 and FS/20/20/34626 (to P. H. Charlton); in part by the European Regional Development Fund Project No. 01.2.2-LMT-K-718-01-0030 (to V. Marozas) under grant agreement with the Research Council of Lithuania; in part by the Estonian Ministry of Education and Research under personal post-doctoral research funding PUTJD815 (to K. Pilt); and in part by the Serbian Ministry of Education, Science and Technological Development Grants 32040 and 41022 (to D. Žikić).

DISCLOSURES

S. Zanelli collaborates with Axelife, a company that designs and develops PPG-based medical devices. D. Kulin is shareholder and employee in E-Med4All Europe Ltd., a Hungarian med-tech startup developing various PPG-based telemedicine solutions. M. Hallab is CEO of Axelife and has authored patents used by Axelife. E. Bianchini is co-founder of QUIPU s.r.l., Pisa, Italy, a spin-off company of the Italian National Research Council and the University of Pisa developing medical software for ultrasound image processing. V. Dittrich is CEO and shareholder of Redwave Medical GmbH, a company developing medical algorithms for pulse wave analysis. None of the other authors has any conflicts of interest, financial or otherwise, to disclose.

AUTHOR CONTRIBUTIONS

P.H.C., B.P., M.B., M.H., V.D., B.H., D.V., D.Ž., and V.M. conceived and designed research; P.H.C., B.P., K.P., M.B., S.Z., D.K., D.Ž., and V.M., performed experiments; P.H.C., B.P., K.P., M.B., S.Z., D.K., D.Ž., and V.M. analyzed data; P.H.C., B.P., K.P., M.B., S.Z., D.K., J.A., D.V., D.Ž., and

Downloaded from journals.physiology.org/journal/ajpheart (080.189.253.250) on March 11, 2022.

Table A4. *Datasets of PPG signals used to assess PPG-derived parameters of vascular age [Modified from (226)]*

Dataset	Reference	Signals	Reference Parameters	No. Subjects	Description
UK Biobank	(224)	PPG	Blood pressure (BP), chronological age	205,337	Single finger PPG waves from middle-aged subjects. The stiffness index, calculated by the PPG device, is also available.
MIMIC	(225)	PPG, BP, electrocardiogram (ECG), others	BP, ankle-brachial index, chronological age	10,000	Recordings from critically ill adults and neonates, lasting from minutes to days. Typically, at finger.
Cuffless BP Estimation	(226, 227)	PPG, ECG	BP	942	Recordings from critically ill patients, each lasting ≥10 min. Extracted from the MIMIC-II Database.
PPG-BP Database	(228)	PPG	BP, chronological age	219	Three finger recordings from adults aged 20–89 with and without cardiovascular disease, three waves per recording.
University of Queensland Vital Signs Dataset	(229)	PPG, ECG, BP	BP	32	Recordings from patients during anesthesia, ranging from minutes to hours in duration.
Pulse Wave Database	(4, 230)	PPG, BP	BP, pulse wave velocity, chronological age, others	4,374(simulated)	Single simulated PPG pulse waves representative of healthy adults aged 25–75.

BP, blood pressure; PPG, photoplethysmogram

V.M. interpreted results of experiments; P.H.C., K.P., M.B. D.Ž., and V.M. prepared figures; P.H.C., B.P., K.P., M.B., S.Z., D.K., J.A., V.D., D.V., D. Ž., and V.M., drafted manuscript; P.H.C., B.P., K.P., M.B., S.Z., D.K., J.A., E.B., C.C.M., D.T.-P., B.H., D.V., D.Ž., and V.M. edited and revised manuscript; P.H.C., B.P., K.P., M.B., S.Z., D.K., J.A., M.H., E.B., C.C.M., D.T.-P., V.D., B.H., D.V., D.Ž., and V.M., approved final version of manuscript.

REFERENCES

1. **Hamczyk MR**, **Nevado RM**, **Barettino A**, **Fuster V**, **Andrés V.** Biological versus chronological aging: JACC focus seminar. *J Am Coll Cardiol* 75: 919–930, 2020. doi:10.1016/j.jacc.2019.11.062.
2. **O'Rourke M**, **Nichols WW**, **Vlachopoulos C.** *McDonald's Blood Flow in Arteries* (6th ed.). London, UK: Hodder Arnold, 2011.
3. **O'Rourke M.** Arterial stiffness, systolic blood pressure, and logical treatment of arterial hypertension. *Hypertension* 15: 339–347, 1990. doi:10.1161/01.HYP.15.4.339.
4. **Charlton PH**, **Mariscal Harana J**, **Vennin S**, **Li Y**, **Chowienczyk P**, **Alastruey J.** Modeling arterial pulse waves in healthy aging: a database for in silico evaluation of hemodynamics and pulse wave indexes. *Am J Physiol Heart Circ Physiol* 317: H1062–H1085, 2019. doi:10.1152/ajpheart.00218.2019.
5. **Vlachopoulos C**, **Aznaouridis K**, **Stefanadis C.** Prediction of cardiovascular events and all-cause mortality with arterial stiffness: a systematic review and meta-analysis. *J Am Coll Cardiol* 55: 1318–1327, 2010. doi:10.1016/j.jacc.2009.10.061.
6. **Bruno RM**, **Nilsson PM**, **Engström G**, **Wadström BN**, **Empana J-P**, **Boutouyrie P**, **Laurent S.** Early and supernormal vascular aging: clinical characteristics and association with incident cardiovascular events. *Hypertension* 76: 1616–1624, 2020. doi:10.1161/HYPERTENSIONAHA.120.14971.
7. **Miyasaka K**, **Shelley K**, **Takahashi S**, **Kubota H**, **Ito K**, **Yoshiya I**, **Yamanishi A**, **Cooper JB**, **Steward DJ**, **Nishida H**, **Kiani J**, **Ogino H**, **Sata Y**, **Kopotic Rj Jenkin K**, **Hannenberg A. Gawande A.** Tribute to Dr. Takuo Aoyagi, inventor of pulse oximetry. *J Anesth* 35: 671–709, 2021. doi:10.1007/s00540-021-02967-z.[34338865]
8. **Sahli D**, **Eliasson B**, **Svensson M**, **Blohmé G**, **Eliasson M**, **Samuelsson P**, **Öjbrandt K**, **Eriksson JW.** Assessment of toe blood pressure is an effective screening method to identify diabetes patients with lower extremity arterial disease. *Angiology* 55: 641–651, 2004. doi:10.1177/00033197040550i605.
9. **Zhang Y**, **Weaver RG**, **Armstrong B**, **Burkart S**, **Zhang S**, **Beets MW.** Validity of Wrist-Worn photoplethysmography devices to measure heart rate: a systematic review and meta-analysis. *J Sports Sci* 38: 2021–2034, 2020. doi:10.1080/02640414.2020.1767348.
10. **The Reference Values for Arterial Stiffness' Collaboration.** Determinants of pulse wave velocity in healthy people and in the presence of cardiovascular risk factors: Establishing normal and reference values. *Eur Heart J* 31: 2338–2350, 2010. doi:10.1093/eurheartj/ehq165.
11. **Allen J**, **Murray A.** Age-related changes in the characteristics of the photoplethysmographic pulse shape at various body sites. *Physiol Meas* 24: 297–307, 2003. doi:10.1088/0967-3334/24/2/306.
12. **Allen J**, **Murray A.** Age-related changes in peripheral pulse timing characteristics at the ears, fingers and toes. *J Hum Hypertens* 16: 711–717, 2002. doi:10.1038/sj.jhh.1001478.
13. **McVeigh GE**, **Bratteli CW**, **Morgan DJ**, **Alinder CM**, **Glasser SP**, **Finkelstein SM**, **Cohn JN.** Age-related abnormalities in arterial compliance identified by pressure pulse contour analysis: aging and arterial compliance. *Hypertension* 33: 1392–1398, 1999. doi:10.1161/01.HYP.33.6.1392.
14. **Hashimoto J**, **Watabe D**, **Kimura A**, **Takahashi H**, **Ohkubo T**, **Totsune K**, **Imai Y.** Determinants of the second derivative of the finger photoplethysmogram and brachial-ankle pulse-wave velocity: the Ohasama study. *Am J Hypertens* 18: 477–485, 2005. doi:10.1016/j.amjhyper.2004.11.009.
15. **Grant MJ**, **Booth A.** A typology of reviews: an analysis of 14 review types and associated methodologies. *Health Info Libr J* 26: 91–108, 2009. doi:10.1111/j.1471-1842.2009.00848.x.
16. **Ouzzani M**, **Hammady H**, **Fedorowicz Z**, **Elmagarmid A.** Rayyan—a web and mobile app for systematic reviews. *Syst Rev* 5: 210, 2016. doi:10.1186/s13643-016-0384-4.
17. **Radha M**, **de Groot K**, **Rajani N**, **Wong CCP**, **Kobold N**, **Vos V**, **Fonseca P**, **Mastellos N**, **Wark PA**, **Velthoven N**, **Haakma R**, **Aarts RM.** Estimating blood pressure trends and the nocturnal dip from photoplethysmography. *Physiol Meas* 40: 025006, 2019. doi:10.1088/1361-6579/ab030e.
18. **Xing X**, **Ma Z**, **Zhang M**, **Gao X**, **Li Y**, **Song M**, **Dong W-FF.** Robust blood pressure estimation from finger photoplethysmography using age-dependent linear models. *Physiol Meas* 41: 025007, 2020. doi:10.1088/1361-6579/ab755d.
19. **Wang Y**, **Chen C**, **Sue C**, **Lu W**, **Chiou Y.** Estimation of blood pressure in the radial artery using strain-based pulse wave and photoplethysmography sensors. *Micromachines* 9: 556, 2018. doi:10.3390/mi9110556.
20. **Cho S I**, **Negishi T**, **Tsuchiya M**, **Yasuda M**, **Yokoyama M.** Estimation system of blood pressure variation with photoplethysmography signals using multiple regression analysis and neural

Downloaded from journals.physiology.org/journal/ajpheart (080.189.253.250) on March 11, 2022.
